# Metabolic regulation of misfolded protein import into mitochondria

**DOI:** 10.1101/2023.03.29.534670

**Published:** 2023-03-29

**Authors:** Yuhao Wang, Linhao Ruan, Jin Zhu, Xi Zhang, Alexander Chih-Chieh Chang, Alexis Tomaszewski, Rong Li

**Affiliations:** 1 Center for Cell Dynamics and Department of Cell Biology, Johns Hopkins University School of Medicine; Baltimore, MD 21205, USA.; 2 Biochemistry, Cellular and Molecular Biology (BCMB) Graduate Program, Johns Hopkins University School of Medicine; Baltimore, MD 21287, USA.; 3 Mechanobiology Institute and Department of Biological Sciences, National University of Singapore; Singapore 117411, Singapore.; 4 Department of Chemical and Biomolecular Engineering, Whiting School of Engineering, Johns Hopkins University; Baltimore, MD 21218, USA.

## Abstract

Mitochondria are the cellular energy hub and central target of metabolic regulation. Mitochondria also facilitate proteostasis through pathways such as the ‘mitochondria as guardian in cytosol’ (MAGIC) whereby cytosolic misfolded proteins are imported into and degraded inside mitochondria. In this study, a genome-wide screen in yeast uncovered that Snf1, the yeast AMP-activated protein kinase (AMPK), inhibits the import of misfolded proteins into mitochondria while promoting mitochondrial biogenesis under glucose starvation. We show that this inhibition requires a downstream transcription factor regulating mitochondrial gene expression and is likely to be conferred through substrate competition and mitochondrial import channel selectivity. We further show that Snf1/AMPK activation protects mitochondrial fitness in yeast and human cells under stress induced by misfolded proteins such as those associated with neurodegenerative diseases.

## Introduction

Mitochondria are vital organelles whose biogenesis and activities in energy production are tightly linked to cellular metabolic control (1,2). Metabolic stress and mitochondrial dysfunction are common factors that drive age-related degenerative diseases such as heart failure and dementia (3,4), and these diseases are often characterized by loss of proteostasis leading to the formation of protein aggregates (3,5,6). The MAGIC pathway, in which cytosolic protein aggregates are concentrated on the surface of mitochondria, disaggregated by molecular chaperones, and then imported to be degraded by mitochondrial proteases, may represent a link between mitochondrial dysfunction and loss of proteostasis (Figure 1—figure supplement 1A) (7–9). Inhibition of this pathway causes prolonged protein aggregation in cytosol after proteotoxic stress (7,8), whereas an elevated burden of MPs in mitochondria can lead to mitochondrial damage, potentially forming a vicious cycle leading to decline in fitness (6). Therefore, understanding how mitochondrial function in facilitating cellular proteostasis is balanced with mitochondrial biogenesis and metabolism may provide key insights into the maintenance of cellular fitness under stresses related to aging.

## Results

### A genetic screening for MAGIC regulators

**Figure 1.**
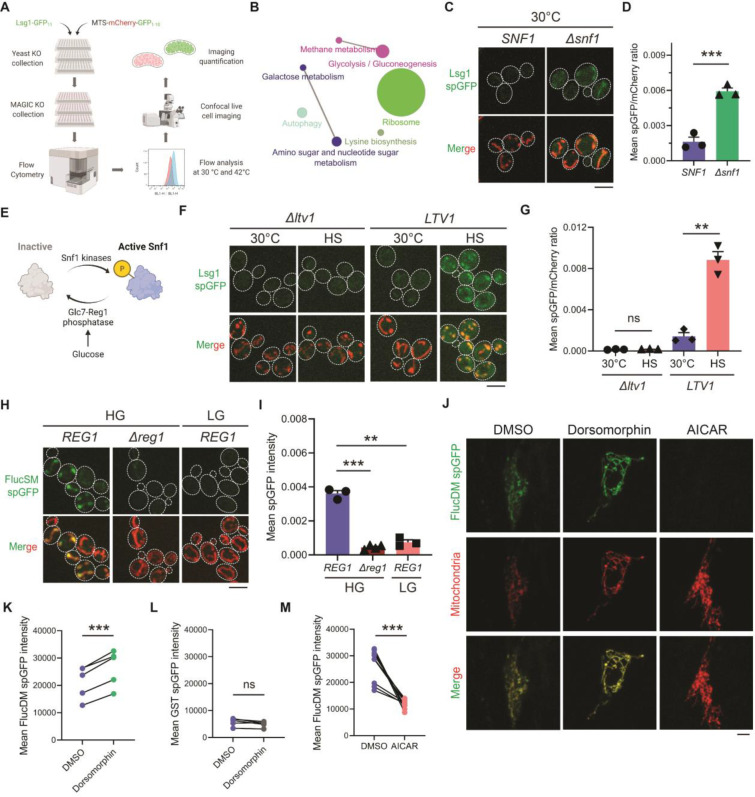
MAGIC regulators revealed by a genome-wide screen in yeast and validations in human RPE-1 cells. (**A**) Workflow of the spGFP-based genetic screen in yeast. (**B**) KEGG pathway analysis of validated mutants that affect MAGIC. The size of the node indicates the number of genes identified. Pathways with at least two associated genes are shown. (**C**, **D**) Representative images (C) and quantification (D) of Lsg1 spGFP signal in WT and *Δsnf1* cells at 30 °C. Shown in (C): Top, Lsg1 spGFP; Bottom, merged images of spGFP and mitochondria labeled with MTS-mCherry. Shown in (D): means ± SEM of spGFP/mCherry ratio. Unpaired two-tailed *t*-test. (**E**) Schematic diagram of Snf1 activation in yeast. (**F**, **G**) Representative images (F) and quantification (G) of Lsg1 spGFP signal in *Δltv1* and wild-type *LTV1* cells at 30 °C and after HS. Shown in (G): means ± SEM of spGFP/mCherry ratio. Paired two-tailed *t*-test. HS: heat shock. (**H**, **I**) Representative images (H) and quantification (I) of FlucSM spGFP in Snf1-inactive (*REG1* in HG) and Snf1-active cells (*Δreg1* in HG; *REG1* in LG). Shown in (H): Top, FlucSM spGFP; Bottom, merged images of spGFP and mitochondria labeled with Tom70-mCherry. Shown in (I): means ± SEM of spGFP intensity. Paired (*REG1* in HG vs. LG) or unpaired (*REG1* vs. *Δreg1* in HG) two-tailed *t*-test. (**J**) Representative images of FlucDM spGFP in RPE-1 cells treated with DMSO, dorsomorphin, or AICAR. Top, FlucDM spGFP; Middle, mitochondria-targeted mCherry; Bottom, merged images. (**K**-**M**) Flow cytometry-based quantifications of FlucDM spGFP in RPE-1 cells treated with DMSO, dorsomorphin, or AICAR (K, M), and GST spGFP in cells treated with DMSO or dorsomorphin (L). Means ± SEM are shown. Paired two-tailed *t*-test. **P < 0.01; ***P < 0.001; ns, not significant, *P* > 0.05. HG: 2% glucose; LG: 0.1% glucose plus 3% glycerol. Scale bars, 5 μm.

To systematically uncover the cellular pathways that influence MAGIC, we performed a high-throughput genetic screen using the non-essential yeast knockout (YKO) collection (10). Lsg1 is an endogenous misfolding-prone protein in cytosol and a known MAGIC substrate (7). To observe the import of Lsg1 and other MPs into mitochondria, we employed the split-GFP (spGFP) reporter, by which a portion of GFP (GFP_1–10_) was targeted into the mitochondrial matrix while the other portion (GFP_11_) was tagged with MPs (Figure 1—figure supplement 1B). Because mitochondrial import requires substrate in an unfolded state (11), globular GFP reconstituted in the cytosol should not be imported. Indeed, spGFP signal of stable cytosolic proteins such as Hsp104 failed to increase after heat shock (7) (Figure 1—figure supplement 1C), whereas mitochondrial spGFP signal of Lsg1 increased significantly after heat shock at 42 °C compared to background at 30 °C (7) (Figure 1F and 1G). The Lsg1 spGFP constructs were introduced into the YKO mutant library, which was first analyzed using high-throughput flow cytometry and then subjected to hits validation using confocal imaging (Figure 1A; Figure 1—figure supplement 1D; details in Materials and Methods).

The validated mutants belonged to two phenotypic classes: 1) 5 mutants whose Lsg1 spGFP signal was significantly greater than the wild type (WT) even without heat shock; and 2) 140 mutants whose Lsg1 spGFP signal did not increase significantly at 42 °C compared to 30 °C (Table S1). KEGG pathway analysis revealed that affected genes encompass many cellular pathways, most notably carbohydrate metabolism and ribosomal biogenesis (Figure 1B). Confocal imaging confirmed that *Δsnf1* cells exhibited an increased spGFP signal compared to WT in the absence of heat stress (Figure 1C and 1D). *SNF1* encodes the yeast homolog of the evolutionarily conserved AMPK and serves as a master energy sensor orchestrating the activation of glucose-repressed gene transcription and the metabolic stress response in glucose-limited conditions (12–14) (Figure 1E). The Class 2 mutants include multiple genes related to ribosomal biogenesis. For example, deletion of *LTV1* that encodes a chaperone required for the assembly of small ribosomal subunits (15) showed only baseline level Lsg1 spGFP fluorescence with no increase at 42 °C (Figure 1F and 1G).

### Snf1/AMPK prevents MP accumulation in mitochondria

To further assess the role of Snf1 in regulating the accumulation of misfolded proteins in mitochondria, we designed a method that specifically imposes proteostasis burden by acute induction of high-level expression of the known MAGIC substrate FlucSM (7,16) tagged with GFP_11_ (FlucSM-GFP_11_) via a β-estradiol-inducible system (17). GFP_1–10_ was stably targeted to the mitochondrial matrix by linking to the matrix protein Grx5 (Grx5-GFP_1–10_). After induction upon β-estradiol addition at normal growth temperature (30 °C) for 90 min, FlucSM spGFP signal increased significantly within mitochondria compared to ethanol control (Figure 1H and 1I; Figure 1—figure supplement 2A–C; Movie S1). The spGFP signal in mitochondria positively correlated with the structural instability of MPs: FlucWT (wild-type luciferase), FlucSM, and FlucDM with the highest structural instability (16) showed an increasing trend of spGFP signal (Figure 1—figure supplement 2D and 2E). We chose to use the intermediate construct, FlucSM-GFP_11_, for testing the effects of modulating Snf1 activity on mitochondrial import of MPs. Deletion of the gene encoding Reg1, which results in constitutive activation of Snf1 in glucose-rich medium (HG: 2% glucose) (18) (Figure 1E), prevented the accumulation of FlucSM spGFP in mitochondria (Figure 1H and 1I). Likewise, wild-type cells that grew in low glucose medium (LG: 0.1% glucose plus 3% glycerol) showed significantly lower FlucSM spGFP compared to cells in HG, and the absence of glycerol in LG (LG-Gly) did not cause any noticeable difference to LG (Figure 1H and 1I; Figure 1—figure supplement 2F and 2G). Snf1 activation under these conditions was validated by the nuclear export of Mig1, which depends on phosphorylation by active Snf1 (19,20) (Figure 1—figure supplement 2H and 2I). In addition, the abundance of FlucSM-GFP_11_ induced by estradiol was not affected by Snf1 activation, and Grx5-GFP_1–10_ level was unchanged in low glucose media and even elevated in *Δreg1* cells - a trend opposite of the spGFP changes (Figure 1—figure supplement 2J). These data exclude the possibility that reduced expression of either protein led to lower spGFP signal in mitochondria. Overall, these results demonstrate that Snf1 activation prevents the accumulation of MPs in mitochondria.

We previously showed that the import of firefly luciferase mutants into mitochondria of human RPE-1 cells was positively correlated with protein instability (7). Using the established spGFP reporter, we found that treatment of cells with dorsomorphin, a chemical inhibitor of AMPK (21), significantly increased mitochondrial accumulation of FlucDM, but not glutathione S-transferase (GST) – a well-folded protein control (Figure 1J–L). In contrast, pharmacological activation of AMPK via 5-aminoimidazole-4-carboxamide ribonucleoside (AICAR) (22), significantly reduced FlucDM accumulation in mitochondria (Figure 1J and 1M). These results suggest that the role of AMPK in the regulation of MP accumulation in mitochondria may be conserved between yeast and human cells.

### Mechanisms of MAGIC regulation by Snf1

**Figure 2.**
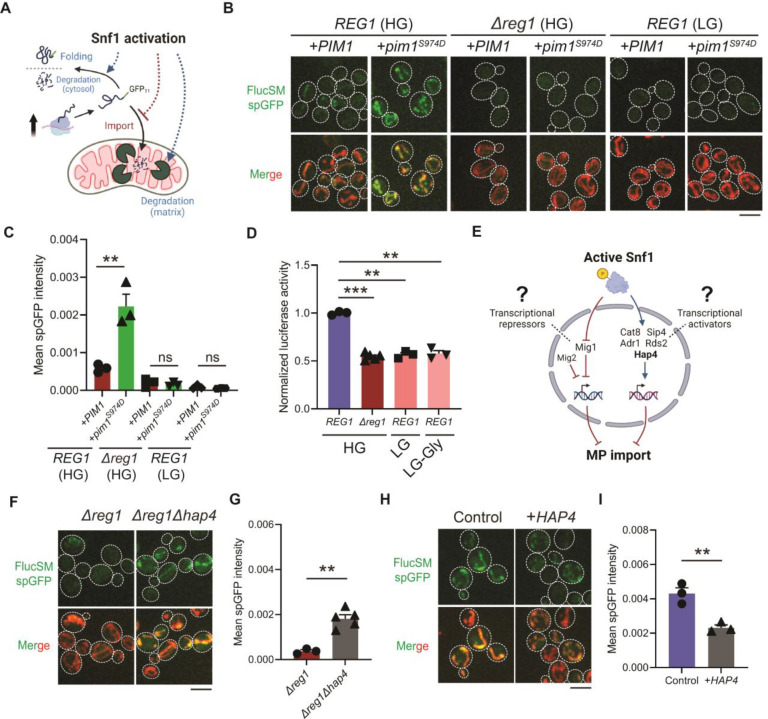
Snf1 negatively regulates mitochondrial import of cytosolic misfolded proteins. (**A**) Schematic diagram showing three possible explanations for reduced spGFP in mitochondria of Snf1-active cells: reduced MPs, blocked import, or enhanced degradation. (**B**, **C**) Representative images (B) and quantification (C) of FlucSM spGFP in Snf1-inactive and Snf1-active cells overexpressing copper-inducible *PIM1* or *pim1*^*S974D*^. Shown in (C): means ± SEM of spGFP intensity. Unpaired two-tailed *t*-test. (**D**) Relative luciferase activity in Snf1-inactive and Snf1-active cells after 90 min of estradiol treatment. Means ± SEM of normalized FlucSM activity are shown. Paired (WT in different media) or unpaired (WT vs. *Δreg1* in HG) two-tailed *t*-test. LG-Gly: 0.1% glucose only. (**E**) Hypothetical regulations of import of MPs through transcriptional repressors and activators downstream of Snf1 activation. (**F**, **G**) Representative images (F) and quantification (G) of FlucSM spGFP in *Δreg1* and *Δreg1Δhap4* cells in HG medium. Shown in (G): means ± SEM of spGFP intensity. Unpaired two-tailed *t*-test. (**H**, **I**) Representative images (H) and quantification (I) of FlucSM spGFP in wild-type cells (control) or with constitutive overexpression of *HAP4* in HG medium. Shown in (I): means ± SEM of spGFP intensity. Unpaired two-tailed *t*-test. **P < 0.01; ***P < 0.001; ns, not significant, *P* > 0.05. Scale bars, 5 μm.

The accumulation of MPs in mitochondria as observed using the spGFP reporter should depend on the relative rates of import versus degradation by mitochondrial proteases, most prominently Pim1 – the conserved Lon protease in yeast (7). Three possible factors could therefore contribute to the reduced accumulation of MPs in mitochondria of Snf1-active cells: 1) enhanced intra-mitochondrial degradation, 2) reduced cytosolic misfolded protein (due to enhanced folding and/or other degradation pathways), and 3) blocked mitochondrial import (Figure 2A). To evaluate the first possibility, an antimorphic mutant *pim1^S974D^* was used to block the degradation of imported FlucSM in the mitochondrial matrix (23). Indeed, in HG medium wild-type cells overexpressing *pim1^S974D^* showed a significantly increased accumulation of FlucSM in mitochondria compared to cells overexpressing *PIM1* (Figure 2B and 2C). However, *pim1^S974D^* overexpression was unable to increase FlucSM accumulation in mitochondria of *Δreg1* cells or WT cells growing in LG medium (Figure 2B and 2C). This result argued against the first possibility, and consistently the abundance of Pim1 protein was not increased by switching to nonfermentable carbon sources (24). To evaluate the second possibility, we used an *in vivo* firefly luciferase assay (25) and assessed the folding of enzymatically active FlucSM after estradiol induction. The result showed that Snf1-active cells exhibited reduced FlucSM luciferase activity, suggesting an increased rather than decreased fraction of misfolded FlucSM (Figure 2D). Furthermore, blocking the activated autophagy pathway in LG medium (26) did not increase FlucSM spGFP in mitochondria (Figure 2—figure supplement 1A and 1B). We thus favor the third possibility that Snf1 activation specifically prevents the import of MPs into mitochondria.

Next, we investigated downstream transcription factors that could mediate the Snf1-regulated MP import (Figure 2E). In the presence of abundant glucose and when Snf1 activity is low, transcriptional repressor Mig1 and its partially redundant homolog Mig2 are localized in the nucleus to confer glucose-repressed gene expression (19,20,27). However, neither single deletion of *MIG1* nor double deletions of *MIG1* and *MIG2* reduced FlucSM spGFP in HG medium (Figure 2—figure supplement 1C and 1D), suggesting that Mig1 and/or Mig2-repressed gene expression was not sufficient to prevent MP import (Figure 2E, left branch). Then we tested if MP import was antagonized by transcriptional activators downstream of Snf1 including Cat8, Hap4, Sip4, Adr1 and Rds2 (13, 28–30) (Figure 2E, right branch). Interestingly, only deletion of *HAP4*, but not other transcriptional activators, significantly rescued FlucSM import defect in *Δreg1* cells with Snf1 activation (Figure 2F and 2G; Figure 2 — figure supplement 1E and 1F). Furthermore, overexpression of Hap4 alone was sufficient to reduce FlucSM spGFP in HG medium (Figure 2H and 2I). These data suggest that Hap4 is a main downstream effector of Snf1 that regulates MP import.

**Figure 3.**
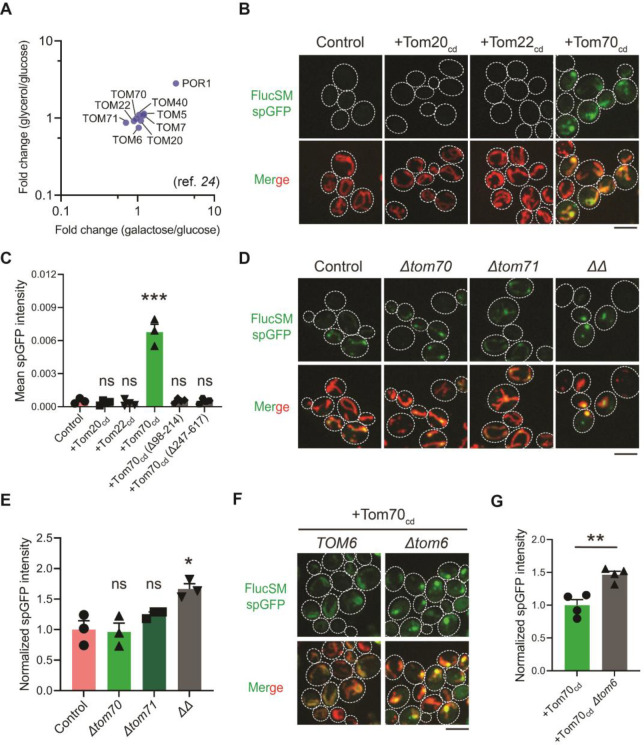
Mechanisms underlying Snf1-regulated MP import into mitochondria. (**A**) Fold changes in protein abundance of TOM complex components in Snf1-active condition (glycerol or galactose) compared to Snf1-inactive condition (glucose). Raw data are retrieved from a published quantitative mass spectrometry dataset (24). (**B**, **C**) Representative images (B) and quantification (C) of FlucSM spGFP in wild-type control cells and cells overexpressing Tom20_cd_, Tom22_cd_, and Tom70_cd_ (C), or truncated Tom70_cd_ variants (Figure 3—figure supplement 1D) in LG medium. Shown in (C) are means ± SEM of spGFP intensity. Unpaired two-tailed *t*-test between control and overexpression strains. (**D**, **E**) Representative images (D) and quantification (E) of FlucSM spGFP in wild-type control, *Δtom70*, *Δtom71*, and *Δtom70 Δtom71* (*ΔΔ*) cells in HG medium. Shown in (D): Top, FlucSM spGFP; Bottom, merged images of spGFP and mitochondria labeled with mCherry-Fis1TM. Shown in (E): means ± SEM of normalized spGFP intensity. Unpaired two-tailed *t*-test. (**F**, **G**) Representative images (F) and quantification (G) of FlucSM spGFP in control and *Δtom6* cells overexpressing Tom70_cd_ in LG medium. Shown in (G): means ± SEM of normalized spGFP intensity. Unpaired two-tailed *t*-test. *P < 0.05; **P < 0.01; ***P < 0.001; ns, not significant, *P* > 0.05. Scale bars, 5 μm.

Hap4 is the transcriptional activation subunit in the Hap2/3/4/5 complex that activates the expression of nuclear encoded mitochondrial proteins and contributes to mitochondrial biogenesis during metabolic shifts or cellular aging (28–32). We hypothesized that elevated expression of mitochondrial preprotein induced by activation of Snf1-Hap4 axis (12,24,32–34) may outcompete MPs for import channels, especially considering that the expression of TOM complex components on the mitochondrial outer membrane was static in Snf1-active cells (24,35) (Figure 3A).

To test this hypothesis, we attempted to restore MP import during Snf1 activation by using high-level expression of the soluble cytosolic domain of import receptors. The cytosolic import receptors lacking membrane-anchoring sequences are known to prevent mitochondrial preproteins from binding TOM complexes and thus inhibit preprotein import (36–38) (Figure 3—figure supplement 1A). Interestingly, overexpression of the cytosolic domain of Tom70 (Tom70_cd_), but not Tom20_cd_ or Tom22_cd_, significantly increased FlucSM import in Snf1-active cells in LG medium (Figure 3B and C). Tom70_cd_ also further increased FlucSM import in HG medium (Figure 3—figure supplement 1B and C). The effect of Tom70_cd_ in cytosol required both the substrate binding and the chaperone-interacting domain (Figure 3C; Figure 3—figure supplement 1D and 1E). It suggests that Tom70-dependent preprotein import may compete with misfolded protein import for limited TOM complexes. To further test if endogenous full-length Tom70 on the mitochondrial outer membrane is dispensable for MP import, we deleted *TOM70* and its paralog *TOM71* and found that in HG medium where mitochondrial respiration is not essential, FlucSM accumulation in mitochondria was not impaired in single mutants and increased in double mutant (Figure 3D and E). This result indicates that MP import does not use Tom70/Tom71 as obligatory receptors. The effect of *Δtom70Δtom71* on MP import was consistent, albeit less pronounced, with Tom70_cd_ overexpression (Figure 3D, E; Figure 3—figure supplement 1B and C). One possibility for the modest effect in double mutant is that given to the functional redundance between Tom20 and Tom70 (39,40), Tom20 receptors in *Δtom70Δtom71* cells could instead mediate preprotein import, whereas cytosolic Tom70_cd_ may have a dominant inhibitory effect on preprotein import by reducing association between preproteins and mitochondrial outer membrane or TOM complexes (36–38). Together, these data suggest that increased expression and receptor-dependent import of normal mitochondrial preproteins during Snf1 activation might indirectly restrict the import of MPs.

As the main entry gate for mitochondrial preproteins, the TOM complex adopts two functional conformations with different substrate specificity: the receptor-free dimer is primarily responsible for importing MIA pathway substrates, and the receptor-bound trimer for Tim23 pathway substrates (41–43). Deletion of Tom6 disassembles the trimer and shifted the conformation equilibrium toward the dimer form (43,44). To test if the substrate selectivity of TOM complex regulates MP import, we eliminated the trimer conformation by deleting *TOM6* and found that it elevated FlucSM import in Snf1-active cells with or without Tom70_cd_ overexpression (Figure 3F and G; Figure 3—figure supplement 1F and G). This result suggests that restricting MP import in Snf1-active cells requires the trimeric TOM complex in addition to the influx of mitochondrial preproteins, and MPs might preferentially cross the mitochondrial outer membrane through the dimeric TOM complex.

### AMPK protects cellular fitness during proteotoxic stress

**Figure 4.**
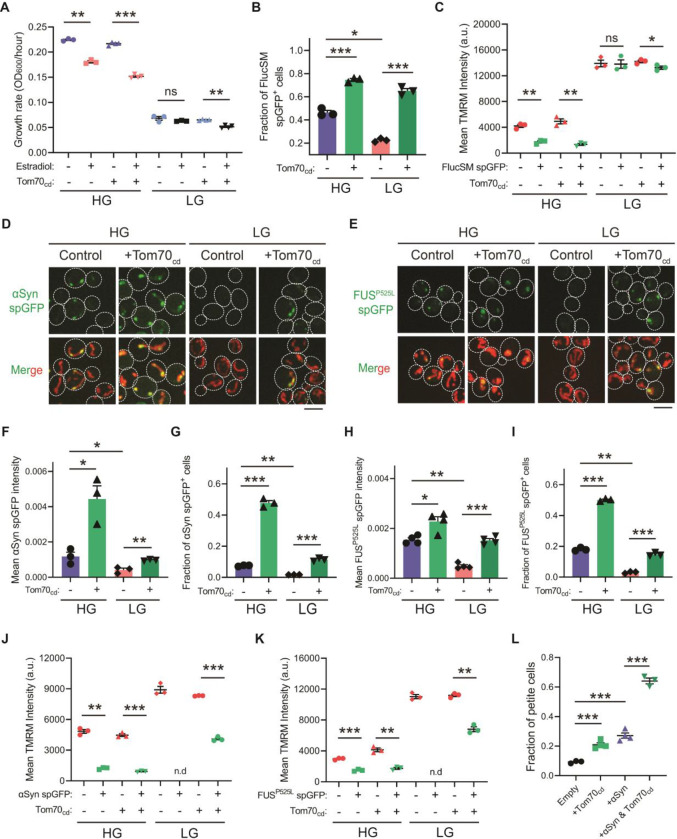
Snf1 activation protests cellular fitness under proteotoxic stress. (**A**) Growth rates of wild-type cells and cells overexpressing Tom70_cd_ with (Estradiol) or without (EtOH) FlucSM expression in HG and LG medium. Means ± SEM of OD_600_ or growth rates are shown. Paired two-tailed *t*-test. (**B**) Fraction of FlucSM spGFP-positive cells measured by flow cytometry. Means ± SEM are shown. Unpaired two-tailed *t*-test for cells growing in the same medium. Paired two-tailed *t*-test for control cells growing in different medium. (**C**) Comparisons of mitochondrial membrane potential between FlucSM spGFP-negative and spGFP-positive cells measured by TMRM. Means ± SEM are shown. Paired two-tailed *t*-test. (**D**-**I**) Representative images and quantifications of α-synuclein (αSyn) spGFP and FUS^P525L^ spGFP signal. Shown in (F, H): means ± SEM of spGFP intensity measured by confocal imaging. Shown in (G, I): means ± SEM of fraction of spGFP-positive cells measured by flow cytometry. Unpaired two-tailed *t*-test for cells growing in the same medium. Paired two-tailed *t*-test for control cells between HG and LG medium. (**J**, **K**) Comparisons of membrane potential between αSyn or FUS^P525L^ spGFP-negative and spGFP-positive cells measured by TMRM. Means ± SEM are shown. Paired two-tailed *t*-test. n.d.: not determined due to limited positive cell counts in control cells growing in LG medium. (**L**) Fraction of respiratory-deficient petite cells measured by using tetrazolium overlay. Means ± SEM are shown. Unpaired two-tailed *t*-test. HG: 2% glucose; LG: 0.1% glucose plus 3% glycerol. *P < 0.05; **P < 0.01; ***P < 0.001; ns, not significant, *P* > 0.05. Scale bars, 5 μm.

We next investigated the physiological effects of metabolic regulation of MAGIC mediated by Snf1/AMPK. Prolonged induction of high-level FlucSM expression imposed a proteotoxic stress and led to a reduced growth rate in HG medium compared to the control, but interestingly no growth reduction was observed under glucose limitation (Figure 4A; Figure 4—figure supplement 1A, 1D and 1E). We reasoned that the lack of growth defect in LG medium could be due to prevention of MP import into mitochondria downstream of Snf1 activation. Supporting this, elevating MP import by Tom70_cd_ overexpression led to a reduced growth rate in LG medium that was dependent on FlucSM expression (Figure 4A; Figure 4—figure supplement 1B). Tom70_cd_ overexpression also exacerbated growth rate reduction due to FlucSM expression in HG medium (Figure 4A; Figure 4—figure supplement 1A). In contrast, negative controls using truncated Tom70_cd_ mutants that could not restore MP import did not produce the same growth defect (Figure 4—figure supplement 1C).

To further test whether the reduction in growth rate during proteotoxic stress was associated with impaired mitochondrial fitness, we assessed mitochondrial membrane potential (MMP) using the dye tetramethylrhodamine methyl ester (TMRM). In HG medium and after 90 min induction of FlucSM, there was a negative relationship between spGFP accumulation and MMP: spGFP-positive cells exhibited a significantly reduced MMP level than spGFP-negative cells (Figure 4C). Again, this difference was not observed in cells that grew in LG, whereas Tom70_cd_ overexpression led to a significant increase in the fraction of spGFP-positive cells with reduced MMP in both HG and LG medium (Figure 4B and C). These results suggest that glucose limitation protects mitochondria and cellular fitness during FlucSM-induced proteotoxic stress through Snf1-dependent inhibition of MP import into mitochondria.

Many neurodegenerative disease-associated aggregation-prone proteins, such as α-synuclein (45), FUS^P525L^ (46,47), TDP-43 (48,49), amyloid beta (50), and C9ORF72-associated poly(GR) dipeptide (51), are detected in mitochondria of human patients or disease models and impair mitochondrial functions. We wonder whether such toxic effects of disease-associated proteins can be counteracted by AMPK activation. First, we used the spGFP reporter in yeast and observed mitochondrial import of α-synuclein and FUS^P525L^ in Snf1-inactive cells under HG medium (Figure 4D and 4E; Figure 4—figure supplement 1F and 1G; Movie S2). We found that Snf1 activation via glucose limitation or *Δreg1* significantly reduced their accumulation in mitochondria, whereas Tom70_cd_ overexpression reversed this effect (Figure 4D–I; Figure 4—figure supplement 1H–K). Mitochondrial import of α-synuclein and FUS^P525L^ in HG medium was associated with lower MMP, and Tom70_cd_ overexpression significantly increased the fraction of spGFP-positive and MMP-low cells in both HG and LG medium (Figure 4G–K). Furthermore, accumulation of α-synuclein in mitochondria correlated with a loss of respiratory capacity, as overexpression of Tom70_cd_ and α-synuclein synergistically promoted the formation of respiration-deficient petite cells (Figure 4L).

**Figure 5.**
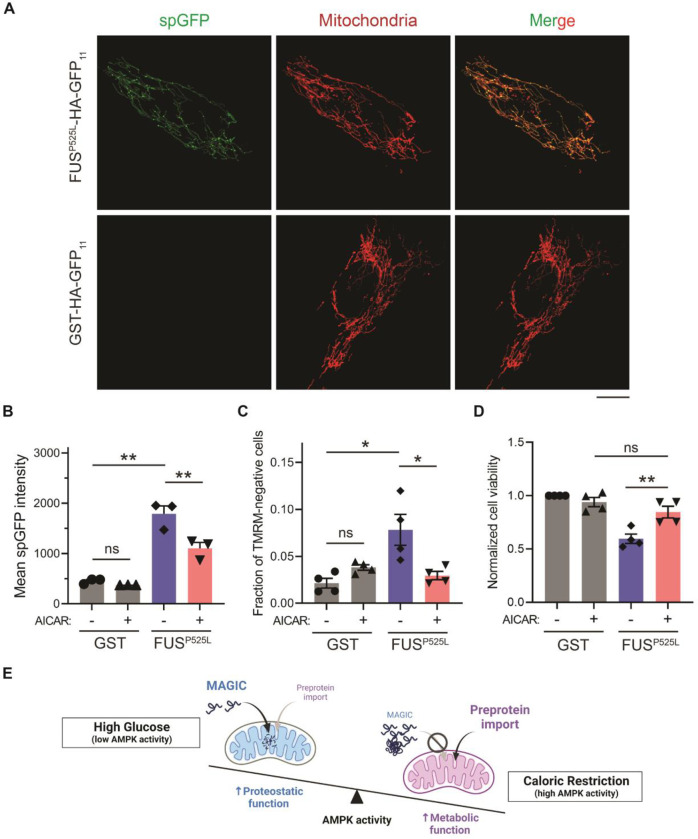
AMPK activation prevents the accumulation of ALS-associated FUS^P525L^ in mitochondria of RPE-1 cells and alleviates FUS-induced cytotoxicity. (**A**, **B**) Representative images (A) and flow cytometry quantification (B) of FUS^P525L^ spGFP and GST spGFP in mitochondria of RPE-1 cells treated with or without AICAR. Shown in (B): Means ± SEM of spGFP intensity. (**C**, **D**) Fraction of TMRM-negative cells (C) and normalized cell viability (D) of RPE-1 cells expressing GST-HA-GFP_11_ or FUS^P525L^-HA-GFP_11_ with or without AICAR treatment. Means ± SEM are shown. (**E**)Working model wherein Snf1/AMPK balances the metabolic and proteostatic function of mitochondria in response to glucose availability. Paired two-tailed *t*-test for the same cell line treated with drug or control medium. Unpaired two-tailed *t*-test between cell lines expressing GST and FUS^P525L^. *P < 0.05; **P < 0.01; ns, not significant, *P* > 0.05. Scale bars, 10 μm.

We next tested whether reducing mitochondrial accumulation of FUS^P525L^ ameliorates its cellular toxicity in human cells. FUS^P525L^ has been shown to bind mitochondrial Hsp60 and ATP synthase β-subunit to induce mitochondrial fragmentation and cell death (46,47). We expressed FUS^P525L^ into human RPE-1 cells by transient transfection and confirmed the entry of FUS^P525L^ into mitochondrial matrix using the spGFP reporter (Figure 5A and 5B). FUS^P525^ expression also caused the loss of MMP and elevated cell death compared to GST control (Figure 5C and 5D). Importantly, mitochondrial accumulation and fitness decline caused by FUS^P525^ expression were significantly reduced by activation of AMPK via AICAR treatment (Figure 5B–D). These results suggest a protective role of AMPK in FUS-induced cellular toxicities possibly through preventing the import of the disease protein into mitochondria.

## Discussion

Metabolic imbalance and loss of proteostasis are interconnected hallmarks of aging and age-related diseases (3,5,52). Various metabolic signaling pathways, such as TOR, AMPK, Sirtuins, and insulin/IGF-1, sense metabolic stimuli, regulate cellular stress responses and influence major cytosolic protein quality control mechanisms including ubiquitin-proteasome pathway and autophagy (52). Mitochondria, the central target of metabolic signaling and major hub of energy production, participate in proteostasis by importing of cytosolic misfolded proteins lacking canonical mitochondrial targeting sequences via the MAGIC pathway (7). Here, we revealed an unexpected link between cellular metabolism and proteostasis through MAGIC by using an unbiased genetic screening in yeast. Our data established Snf1/AMPK as a key regulator of misfolded protein import, which balances the mitochondrial metabolic and proteostatic functions in response to glucose availability and protects mitochondrial fitness under proteotoxic (Figure 5E). We speculate that, when glucose level is high and cells rely on glycolysis for ATP production, mitochondria play a ‘moonlighting role’ in cellular proteostasis through MAGIC, a process dependent on mitochondrial import and proteostasis machineries including chaperones, translocons, and proteases (7). On the other hand, when glucose is limited and cells rely on oxidative phosphorylation for ATP generation, AMPK activation shuts down MAGIC and promotes import of essential mitochondrial preproteins, thus ensuring mitochondrial fitness and energy production.

The downstream mechanism of this regulation remains to be fully elucidated. We propose that in yeast Snf1 activates the Hap4-dependent expression of mitochondrial preproteins which could compete with MPs for limited TOM complexes under glucose-limiting conditions. However, since Snf1/Hap4 activation systematically elevates the expression of hundreds of mitochondrial preproteins (24,32–35), it remains to be determined if specific preproteins or cytosolic factors are directly involved in inhibiting MP import. In addition, the trimeric form of the TOM complex maintained by Tom6 is important for limiting MP entry under glucose restriction. Although the exact mechanism of action is unknown, we speculate that the receptor-binding state and substrate selectivity of different TOM conformations (43) could affect the permeability for MPs to enter mitochondria. Existing proteomic data suggest that the abundance of Tom6 is unaffected by Snf1 activation (24) (Figure 3A). As Tom6 can be phosphorylated by Cdk1 in a cell-cycle dependent manner (44), it is important to investigate if Tom6 or other TOM complex components are targets of Snf1 kinase activity to directly modulate substrate specificity of the TOM complex.

An interesting question raised in this study is whether MAGIC is beneficial or detrimental to cells. Our data suggest that under physiological stress-free conditions, MP import and degradation in mitochondria is well-tolerated, but an acute or chronic increase in the cytosolic misfolded protein load could overwhelm mitochondrial proteostasis capacity leading to organellar damage. If so, the regulation of MAGIC by AMPK could help explain the beneficial effect of caloric restriction on life span extension in model organisms (32,53). In human, the role of AMPK in health and diseases are complex and not fully understood (53–55). While AMPK activity and mitochondrial gene expression via downstream transcriptional factors such as PGC-1α and FOXO are elevated during health-benefitting activities such as exercise (56), hyperactivated AMPK has also been reported in several neurodegenerative diseases with proteostasis decline (54). Our findings suggest that elevating AMPK activity may be beneficial in alleviating proteotoxicity associated with degenerative diseases. Further studies using genetic approaches and relevant *in vivo* models could help elucidate the physiological role of AMPK in balancing proteostasis and mitochondrial fitness.

## Materials and Methods

### Yeast strains, plasmids and culture media

Yeast strains used in this study are based on the BY4741 strain background and listed in Table S2. Gene deletion and protein tagging were performed through PCR-mediated homologous recombination (57) and verified by PCR genotyping. MAGIC YKO collection was constructed by incorporating MTS-mCherry-GFP_1–10_ under GPD promoter into the TRP1 locus and tagging endogenous Lsg1 with GFP_11_ in the YKO collection (10). Human α-synuclein tagged with GFP11 under GPD promoter was cloned and inserted into the *ura3Δ0* locus. FlucSM-HA-GFP_11_ and FUS^P525L^-HA-GFP_11_ under GAL1 promoter were cloned from plasmids from our previous study (7) and plasmid 416Gal-FUS-P525L-YFP, a gift from Aaron Gitler (Addgene plasmid #29628). FlucWT-HA-GFP_11_ and FlucDM-HA-GFP_11_ plasmids were constructed using site-directed mutagenesis kit (NEB) based on FlucSM-HA-GFP_11_. Both GFP_11_-tagged Fluc proteins and GEM transcriptional factor (cloned from pJW1663, Addgene plasmid #112037) were stably integrated into yeast genome. GFP_1–10_ was fused with the mitochondrial matrix protein Grx5 under GPD promoter, except in experiments involving *PIM1* or *pim1^S974D^* mutant and α-synuclein spGFP where GFP_1–10_ was fused to the C-terminus of endogenous Grx5 to avoid signal saturation. Wild-type *PIM1* or *pim1^S974D^* mutant under CUP1 promoter, *HAP4*, cytosolic domain of Tom20 (1–97 aa), Tom22 (38–617aa), Tom70 (38–617aa) and truncated variants of Tom70cd under GPD promoter were cloned and stably integrated into yeast genome. Mitochondrial outer membrane was labeled with Tom70-mCherry or Tom70-RFP, except for the Tom70/71 deletion experiments in which mitochondria were labeled with mCherry-Fis1TM (9).

MAGIC YKO library construction, flow cytometry, and imaging during high-throughput screen were performed with synthetic defined minus histidine (SD-His) medium. Synthetic complete (SC) supplemented with 2% glucose (HG), 0.1% glucose plus 3% glycerol (LG), or 0.1% glucose (LG-Gly) was used for confocal imaging, luciferase assays, biochemistry, and TMRM staining. YEP medium (yeast extract-peptone) supplemented with 2% glucose (HG) or 0.1% glucose plus 3% glycerol (LG) was used for growth assays. Optical density at 600 nm (OD_600_) was used to estimate the amount of yeast cells used in the various experiments.

### Drug treatments

β–estradiol (E2758, MilliporeSigma) was dissolved in H_2_O and added to a final concentration of 1 μM. CuSO_4_ (C1297, MilliporeSigma) was dissolved in H_2_O and added to a final concentration of 0.5 mM. D-luciferin potassium salt (LUCK, GoldBio) was freshly dissolved in appropriate yeast media to a final concentration of 0.5 mM. Dorsomorphin (S7840, Selleck Chemicals; 11967, Cayman Chemical) dissolved in DMSO was added to RPE-1 cells at the final concentration of 10 μM for 24 hours (58). AICAR was dissolved in DMSO (S1802, Selleck Chemicals) and added to the final concentration of 2 mM for 48 hours in the FlucDM experiment, or dissolved directly in media at the concentration of 2 mM (10010241, Cayman Chemical) for the FUS^P525L^ experiment (59).

### Yeast library construction and genome-wide screen

MAGIC YKO was constructed with a 2-step transformation using the Frozen-EZ Yeast Transformation II Kit (T2001, Zymo Research) following the microscale protocol in 96-well format. First, knockout strains were grown to saturation in deep-well plates containing 1 ml of YPD broth with G418 (200 μg/mL, Corning). 150 μL of refreshed mid-log phase cultures and 0.2 μg of MTS-mCherry-GFP_1–10_-clonNat DNA were used in the transformation setup on the epMotion 5075 liquid handling workstation (Eppendorf). To optimize transformation efficiency, the transformation mixtures were incubated for 2 hours and at the end of transformation they were transferred into deep-well plates with 4 volumes of YPD for 2 hours of outgrowth at 30 °C. The transformants were selected for 4–5 days in 1 ml of YPD broth with clonNAT (200 μg/mL, GoldBio), resulting in the intermediate MTS-mCherry-GFP_1–10_-clonNat library. Then the Lsg1-HA-GFP_11_ tagging PCR product was integrated into the genome of the intermediate strains following the same protocol, with the exception that the finial library was selected in SD-His medium.

Total 4645 YKO strains with Lsg1 spGFP reporter were cultured in 96-well plates, and spGFP intensities before and after heat shock (30 min at 42 °C) were measured at 488 nm excitation with appropriate filters on Attune NxT flow cytometer equipped with an auto sampler (Thermo). After subtracting background from the populational mean spGFP intensity, KOs displaying different spGFP pattern were determined by a cutoff (smaller than 1.1-fold increase after heat shock) and further validated by live cell confocal imaging. Based on the phenotype of mitochondrial spGFP intensity of each mutant at two imaging time points, Class 1 mutants were determined by the *P* value of comparing the spGFP/mCherry ratio of each single cell between KO and WT at permissive temperature, *P* < 0.01, and Class 2 mutants were determined by the *P* value of comparing the spGFP intensity of each single cell of before and after heat shock for the same mutant, *P* > 0.01. Genes involved in known mitochondrial import pathways were excluded from analysis.

### Confocal microscopy and imaging conditions

Live cell images were acquired using a Yokogawa CSU-10 spinning disc on the side port of a Carl Zeiss 200 m inverted microscope or a Carl Zeiss LSM-780 confocal system. Laser 488 or 561 nm excitation was applied to excite GFP or mCherry, respectively, and the emission was collected through the appropriate filters onto a Hamamatsu C9100–13 EMCCD on the spinning disc confocal system or the single-photon avalanche photodiodes on the Zeiss 780 system. Regarding the multi-track acquisition, the configuration of alternating excitation was used to avoid the bleed-through of GFP (for dual color imaging, GFP or mCherry labeled controls were applied for laser and exposure settings). The spinning disc and the LSM780 were equipped with a 100×1.45 NA Plan-Apochromat objective and a 63×1.4 oil Plan-Apochromat objective, respectively. For yeast 3D imaging, 0.5 μm step size for 6 μm in total in Z; for human cells, 1 μm step size. Images were acquired using MetaMorph (version 7.0, MDS Analytical Technologies) on the CSU-10 spinning disc system and Carl Zeiss ZEN software on the LSM780.

Yeast culture condition for imaging: yeast cells were cultured in SC or SD-His with appropriate carbon source overnight at 30 °C. The cells were then refreshed in the corresponding medium for at least 3 hours at 30 °C until reaching an OD_600_ of about 0.2. For estradiol-GEM inducible systems, 1 μM of β–estradiol was added to the medium for 90 min unless indicated otherwise. For copper-inducible overexpression of *PIM1* or its mutant, 0.5 mM CuSO_4_ was added for 2 hours, followed by the estradiol induction for 2 hours. All images in the same experiments were acquired with the same laser and exposure settings. Image processing was performed using ImageJ software (NIH) or Imaris software (Oxford Instruments Group). For visualization purposes, images were scaled with bilinear interpolation and shown as the maximum projection on Z for fluorescent channels. Cell boundaries were delineated according to white-field images.

### Split-GFP quantification

Split-GFP fluorescence from confocal images was quantified by using a custom Python code described before (7). In brief, mCherry and GFP intensities were summed along the z-axis, and then subjected to a random walk segmentation of the background and watershed segmentation of adjoining cells. For each cell, the mCherry channel was thresholded at 5% of maximal value to detect mitochondria, and median GFP intensity within mitochondria was calculated as spGFP intensity per cell. In the YKO imaging validation, Lsg1 spGFP/mCherry ratio of each cell was used for statistical analyses. For Lsg1 spGFP signal detected in *Δsnf1*, *Δltv1*, and *WT* cells, populational means spGFP/mCherry of at least 3 biological repeats were calculated. Adjusting Lsg1 spGFP intensity to mitochondrial mCherry intensity avoided the potential effect of changing local abundance of GFP_1–10_ on Lsg1 spGFP signal after heat shock. For estradiol-inducible systems that did not involve heat shock, populational mean spGFP intensity of each biological repeat was used for the following analyses. For the flow cytometry quantification, populational mean GFP intensities of at least 25,000 single cells were calculated for the following analyses. Most quantifications were shown as absolute intensity values with an arbitrary unit. Normalized spGFP intensities were calculated to highlight the relative changes.

### Mammalian cell culture, transfection, imaging and quantification

Human RPE-1 cells were cultured in Dulbecco’s Modified Eagle Medium: Nutrient Mixture F-12 (DMEM/F12) (GIBCO), supplemented with 10% (v/v) fetal bovine serum (FBS), 100 IU/ml penicillin. Transient transfections were performed with Lipofectamine 3000 (Invitrogen) according to the manufacturer’s instructions. RPE-1 cells were dually transfected with MTS-mCherry-GFP_1–10_ and the protein of interest tagged with GFP_11_ (2.5 μg of each plasmid was applied). For imaging, MatTek (P35G-0-14-C) dish was used to culture cells, and cells were located using the mCherry channel only. Cells were imaged or analyzed by flow cytometry after 24 or 48 hours of transfection for FUS^P525L^ or FlucDM, respectively. For flow cytometry analysis of FUS^P525L^ spGFP system, cells were permeabilized with digitonin buffer (0.32 M sucrose, 5 mM CaCl_2_, 3 mM Mg[Acetate]_2_, 0.1 mM EDTA, 10 mM Tris-HCl, 100 ug/ml digitonin) for 8–10 min, in order to remove spGFP signal outside of mitochondria in cytosol.

To evaluate cell death caused by FUS^P525L^ overexpression, equal number of RPE-1 cells were seeded in 6-well plates and transfected with GST or FUS^P525L^, with or without AICAR. Compared to GST transfection control, FUS^P525L^ resulted in significant floating dead cells. Number of attached cells after 24 hours of transfection were analyzed with Attune NxT flow cytometer as a proxy for cell viability.

### Cell lysates, immunoblots and antibodies

For yeast experiments, 1–2 ml of yeast cells in the indicated background and medium was collected by centrifugation and snap frozen in liquid nitrogen for storage. Pellets were disrupted, boiled in 120 μl 1X LDS sample buffer (Thermo) for 10 min, and vortexed with an equal volume of 0.5 mm acid-washed glass beads to break cells at 4 °C for 2 min with a 1 min interval. Cell lysates were boiled for 5 min, separated from glass beads by 15,000 *g* centrifugation at room temperature for 30 sec, and analyzed by SDS-PAGE. For mammalian data, RPE-1 cells were washed with PBS and lysed with RIPA buffer (MilliporeSigma) supplemented with protease inhibitors on ice for 20–30 min. Cell lysates were further sonicated and incubated on ice for 5 min, followed by 10 min 21,200 *g* centrifugation at 4 °C. The supernatant was collected and analyzed by SDS-PAGE.

Transfer was performed using iBlot2 (Thermo) and immunoblots were developed using Clarity Western ECL substrate (Bio-Rad) for HRP-linked secondary antibodies, or directly using fluorescent IRDye secondary antibodies (LI-COR). Data were acquired by using LI-COR imaging systems and analyzed in Image Studio (LI-COR). HA-tag (C29F4) rabbit mAb #3724 from Cell Signaling Technology. PGK1 mouse mAb (22C5D8) from Invitrogen. FLAG mouse clone M2 (F1804) from MilliporeSigma. GFP Living Colors A.v. mAb clone JL-8 (632381) from Takara Bio.

### Firefly luciferase assays

Firefly luciferase assays in yeast were carried out as described previously (24). In brief, after 90 min of estradiol induction, 100 μl of cells was vigorously mixed with 100 μl of 1 mM D-luciferin in a white 96-well plate (655073, Greiner Bio-One), and light emission was immediately measured by the luminescence detection mode in Cytation 5 (Biotek). Luciferase activities were normalized to cell density measured by OD_600_ and adjusted to total abundance of FlucSM protein measured by immunoblotting.

### Mig1 nucleocytoplasmic translocation

The nucleocytoplasmic distribution of Mig1-GFP was quantified using a custom ImageJ macro and MATLAB script as described previously (60). In brief, nuclear protein Pus1-RFP was used to create nucleoplasmic mask for each cell (61). Cytoplasm was defined by a dilated nuclear mask (59). The nuclear-cytoplasmic ratio of each cell was calculated by dividing the mean nuclear intensity by the mean cytoplasmic intensity. Populational means nuclear-cytoplasmic ratio of at least 3 biological replicates were used for statistical analyses.

### Yeast growth curve

Yeast cells with indicated genetic background were cultured in corresponding media. Overnight cultures were refreshed for 4 hours at 30 °C and the OD_600_ of the cells was measured and adjusted to 0.05. Diluted cell suspension was added to a 96-well plate with 2 μM estradiol or ethanol as control. The wells along the perimeter of the plate were pre-filled with 200 μL cell-free medium to prevent evaporation. The OD_600_ was continuously monitored at 30 °C using Citation 5 (Biotek) every 20 min with constant shaking. Data were extracted and analyzed using the R package GroFit (https://cran.r-project.org/src/contrib/Archive/grofit/) (62).

### Mitochondrial membrane potential measurements

Yeast cells expressing MPs and growing in appropriate medium was collected, incubated with 2.5 μM TMRM (21437, Cayman Chemical) for 15 min at 30 °C and washed twice by fresh medium before recording with Attune NxT flow cytometer equipped with appropriate filter sets. A SpGFP intensity threshold was applied so that less than 1% of cells displayed positive spGFP in the ethanol-treated control groups with no expression of MPs. Mean TMRM intensities of at least 25,000 cells were calculated for each biological replicate.

RPE-1 cells transfected with either GST or FUS^P525L^ for 24 hours were washed once with PBS and added with complete media containing 150 nM TMRM for 30 min at 37 °C. After incubation, cells were washed with PBS and trpsonized into single cells. Cell suspensions were pelleted and re-suspended in PBS for analysis on the Attune NxT flow cytometer.

### Tetrazolium overlay assay

Yeast tetrazolium overlay was performed to measure the respiratory deficiency in a yeast population as previously described (63). In brief, yeast cells were inoculated in YPD media at 30 °C overnight. Around 100 cells were plate on YPD plates and grew for 4 days at 30 °C. The tetrazolium test medium consists of 1.5% agar and 0.1% tetrazolium (17342, Cayman Chemical) in 0.067 M phosphate buffer at pH 7.0 (63). Test was performed by pouring 15 ml of melted test medium at 55 °C over a YPD plate. The number of large red colonies (respiration-sufficient) and small white colonies (respiration-deficient) were counted after 1 hour of incubation at 30 °C.

### Super resolution imaging

Structured illumination microscopy (SIM) images were acquired with a GE OMX-SR Super-Resolution Microscope 3D Structure Illumination (3D-SIM) equipped with high-sensitivity PCO sCMOS cameras, or LSM880-Airyscan FAST Super-Resolution microscopy equipped with 63×/1.4 PlanApo oil. GFP and mCherry were excited with 488 nm and 568 nm lasers, respectively. AThe SIM images were reconstructed with the Softworx and aligned following the Applied Precision protocols, and Zeiss images were reconstructed with Airyscan processing. 3D rendering was performed with Imaris (Oxford Instruments Group).

### Statistical analysis

Descriptions of statistical tests and *P* values can be found in Figure Legends. At least three biological replicates were analyzed in all experiments. Statistical analyses were performed with GraphPad Prism 6.0. No statistical methods were used to predetermine the sample size. The experiments were not randomized, and the investigators were not blinded to allocation during experiments and outcome assessment.

## Supplementary Material

Supplement 1
