# Supplementary material for: Metabolic regulation of misfolded protein import into mitochondria
Source: bioRxiv. 2023 Aug 31:2023.03.29.534670. Originally published 2023 Mar 29. Preprint. [Version 2] doi: 10.1101/2023.03.29.534670 (PMC10081186; doi:10.1101/2023.03.29.534670)
Supplement: Supplement 5 [file NIHPP2023.03.29.534670v2-supplement-5.pdf]

bioRxiv preprint doi: https://doi.org/10.1101/2023.03.29.534670; this version posted August 31, 2023. The copyright holder for this preprint (which was not certified by peer review) is the author/funder, who has granted bioRxiv a license to display the preprint in perpetuity. It is made available under aCC-BY 4.0 International license.

Supplementary Materials

Table S1: List of validated MAGIC regulators

Table S2: List of yeast strains and plasmids

Movie S1: 3D reconstructed SIM images showing FlucSM spGFP inside mitochondria after 90 min estradiol treatment. Mitochondrial outer membrane is labeled with Tom70-mCherry.

Movie S2: 3D reconstructed SIM images showing FUS^{P525L} spGFP inside mitochondria after 100 min estradiol treatment. Mitochondrial outer membrane is labeled with Tom70-mCherry.

Figure 1—figure supplement 1

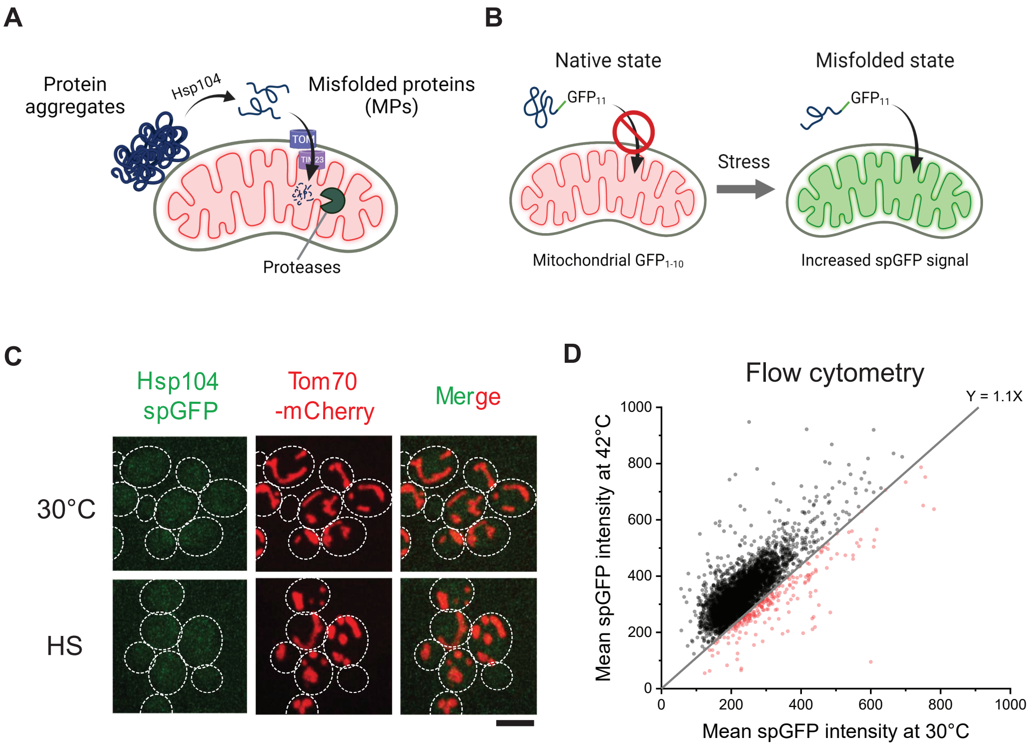

bioRxiv preprint doi: https://doi.org/10.1101/2023.03.29.534670; this version posted August 31, 2023. The copyright holder for this preprint (which was not certified by peer review) is the author/funder, who has granted bioRxiv a license to display the preprint in perpetuity. It is made available under aCC-BY 4.0 International license.

Figure 1—figure supplement 1. Schematics of MAGIC pathway and spGFP-based imaging in the whole-genome screen in yeast. (**A**) Schematic diagram of MAGIC involving the import of cytosolic misfolded proteins through mitochondrial import machineries and the subsequent degradation under proteotoxic stresses (heat shock or overload of MPs). (**B**) Schematic diagram of spGFP reporter. (**C**) Hsp104 spGFP in mitochondria at 30 °C and after heat shock (HS) at 42 °C for 30 min. Endogenous Hsp104 was tagged with GFP_{11}, while $GFP_{1\text{-}10}$ was constitutively targeted to the mitochondrial matrix by linking to a matrix protein Grx5. Similar results were obtained by using MTS-mCherry-$GFP_{1\text{-}10}$ (*10*). 3 biological repeats, 63 cells (30 °C) and 220 cells (HS) imaged. Scale bar, 5 µm. (**D**) Flow cytometry readouts of yeast knockout mutants at 30 °C and after 42 °C HS. Red dots represent mutants that failed to show an increase in Lsg1 spGFP after HS.

Figure 1—figure supplement 2

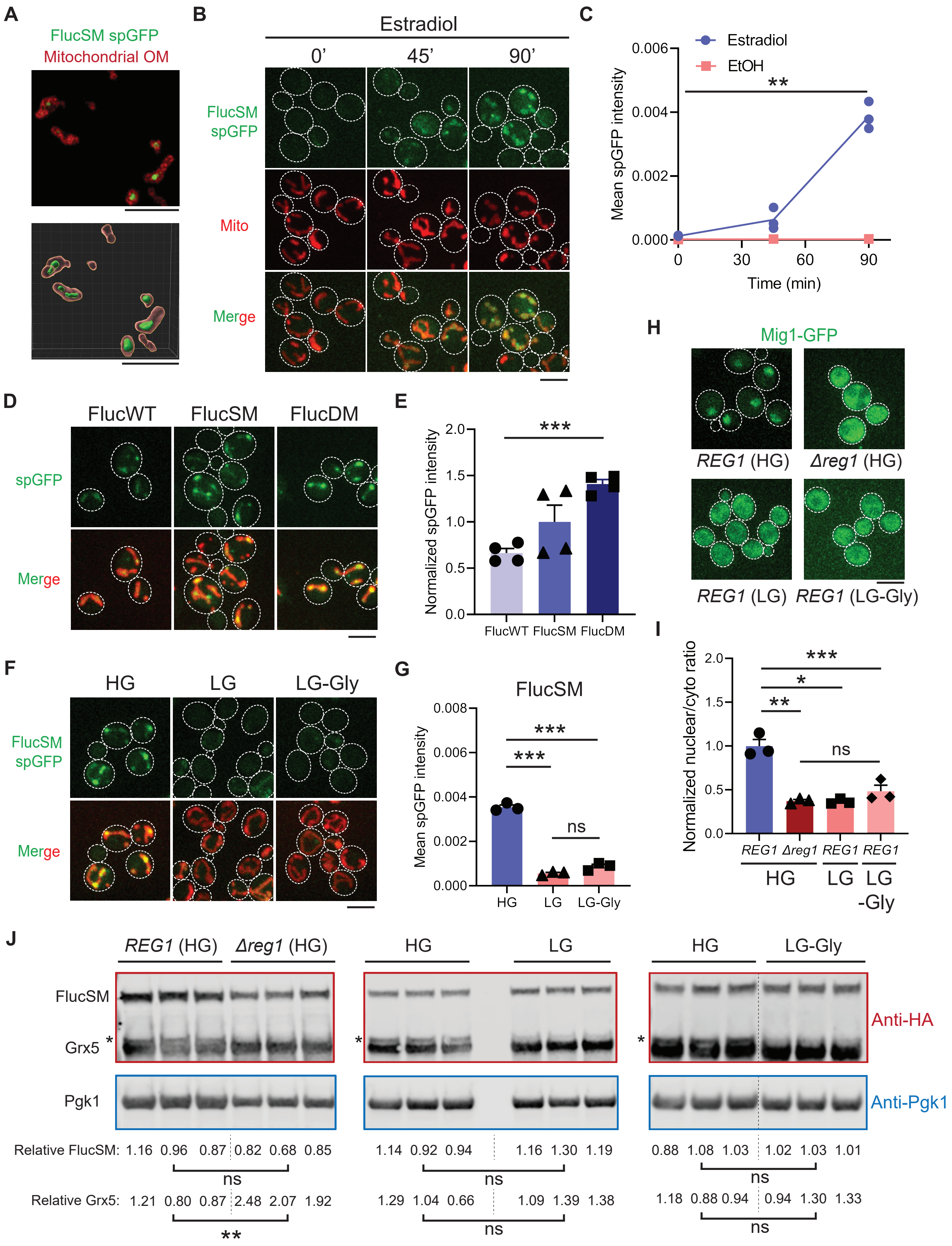

bioRxiv preprint doi: https://doi.org/10.1101/2023.03.29.534670; this version posted August 31, 2023. The copyright holder for this preprint (which was not certified by peer review) is the author/funder, who has granted bioRxiv a license to display the preprint in perpetuity. It is made available under aCC-BY 4.0 International license.

Figure 1—figure supplement 2. Snf1 regulates the accumulation of misfolded proteins in mitochondria after acute overexpression of FlucSM. (**A**) Representative super-resolution imaging for FlucSM spGFP signal in mitochondria after 90 min estradiol induction. Top, maximum projection. Bottom, 3D rendering. OM: outer membrane labeled by Tom70-mCherry. (**B**, **C**) Representative images (B) and quantification (C) of time-dependent accumulation of FlucSM spGFP signal in mitochondria at 30 °C after estradiol or ethanol treatment in HG medium. Shown in (B): Top, FlucSM spGFP; Middle: mitochondria labeled with Tom70-mCherry; Bottom: merged images. Shown in (C): means ± SEM of spGFP intensity. Paired two-tailed *t*-test comparing 0 min and 90 min estradiol treatment. (**D**, **E**) Representative images (D) and quantification (E) of FlucWT, FlucSM, and FlucDM spGFP after 90 min estradiol treatment in HG medium. Shown in (E): means ± SEM of normalized spGFP intensities. Unpaired two-tailed *t*-test between FlucWT and FlucDM. (**F**, **G**) Representative images (F) and quantification (G) of FlucSM spGFP in cells that grew in HG, LG, and LG-Gly media. Shown in (G): means ± SEM of spGFP intensity. Paired two-tailed *t*-test. (**H**, **I**) Representative images (H) and quantification (I) of the nuclear-cytoplasmic translocation of Mig1-GFP. Shown in (I): means ± SEM of normalized Mig-GFP nuclear-cytoplasmic ratio. Paired (WT in HG vs. LG or LG-Gly) or unpaired (WT vs. *Δreg1* in HG) two-tailed *t*-test, and one-way ANOVA for comparing *Δreg1* in HG, WT in LG, and WT in LG-Gly). (**J**) Immunoblots of FlucSM-HA-GFP_{11} and Grx5-HA-$GFP_{1\text{-}10}$ in cell lysates. Relative FlucSM levels (FlucSM/Pgk1) and relative Grx5 levels (matured Grx5/Pgk1) of three biological repeats are shown. Paired (HG vs. LG or LG-Gly) or unpaired (WT vs. *Δreg1* in HG) two-tailed *t*-test. Asterisks indicate the precursor species of Grx5-HA-$GFP_{1\text{-}10}$ that are excluded from quantification. HG: 2% glucose; LG: 0.1% glucose plus 3% glycerol; LG-Gly: 0.1% glucose. EtOH: ethanol. *$P < 0.05$; **$P < 0.01$; ***$P < 0.001$; ns, not significant, $P > 0.05$. Scale bars, 5 μm.

Figure 1—figure supplement 3

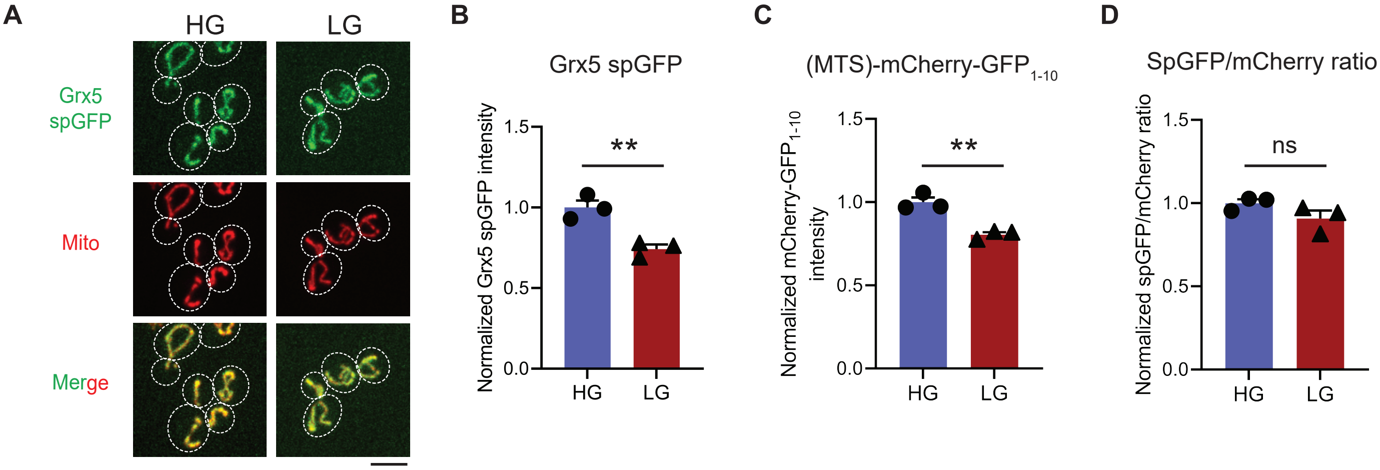

bioRxiv preprint doi: https://doi.org/10.1101/2023.03.29.534670; this version posted August 31, 2023. The copyright holder for this preprint (which was not certified by peer review) is the author/funder, who has granted bioRxiv a license to display the preprint in perpetuity. It is made available under aCC-BY 4.0 International license.

Figure 1—figure supplement 3. **Snf1 activation only modestly affects spGFP reconstitution.** (**A-D**) Representative images (A) and quantification of Grx5 spGFP (B), MTS-mCherry-GFP1-10 (C) and spGFP-to-mCherry ratio (D) in HG or LG medium. Means ± SEM are shown in (B-D). Unpaired two-tailed *t*-test. ***P* < 0.01; ns, not significant, *P* > 0.05. HG: 2% glucose; LG: 0.1% glucose plus 3% glycerol. Scale bars, 5 μm.

Figure 2—figure supplement 1

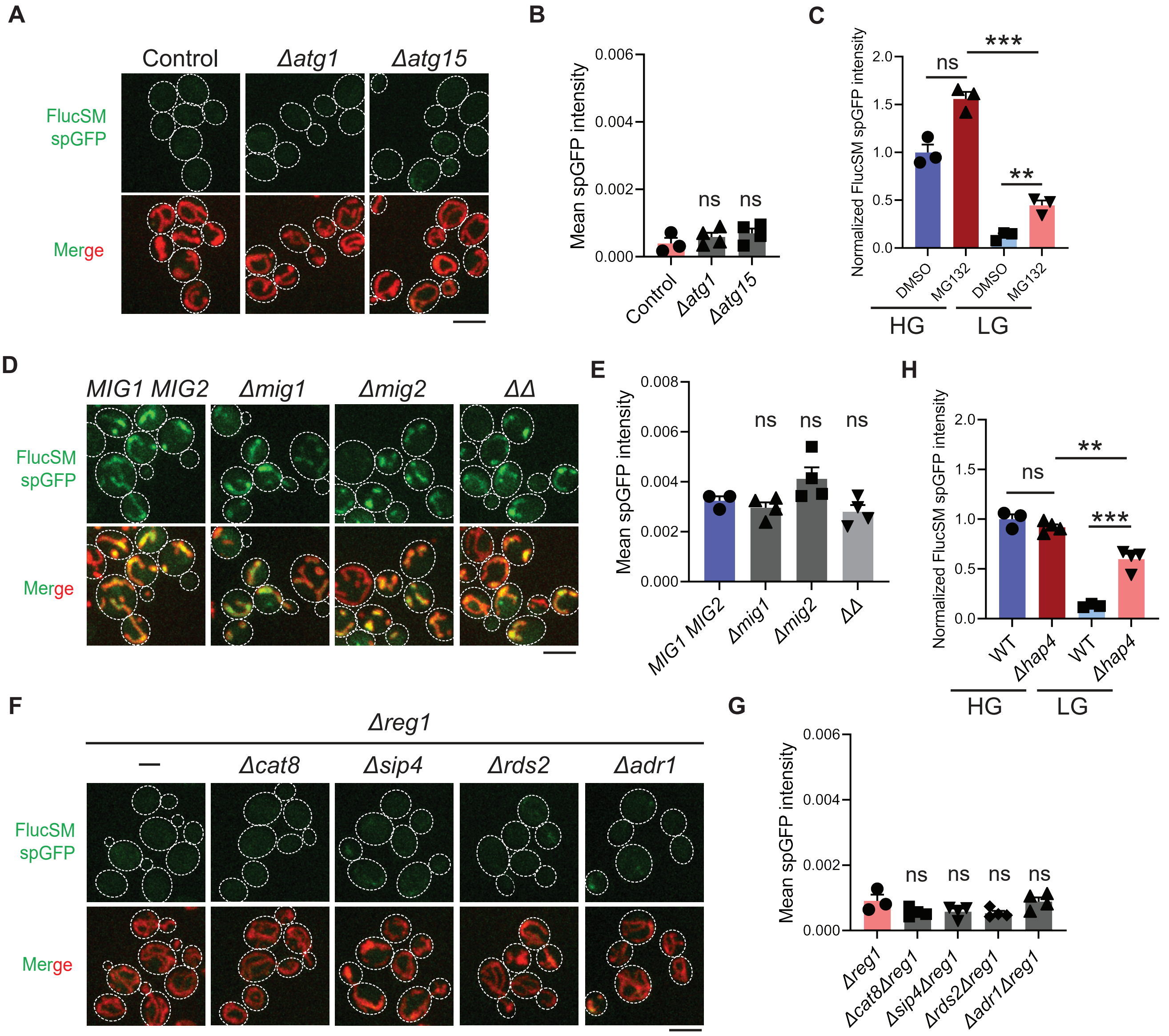

bioRxiv preprint doi: https://doi.org/10.1101/2023.03.29.534670; this version posted August 31, 2023. The copyright holder for this preprint (which was not certified by peer review) is the author/funder, who has granted bioRxiv a license to display the preprint in perpetuity. It is made available under aCC-BY 4.0 International license.

Figure 2—figure supplement 1. Reduced accumulation of misfolded proteins in mitochondria under Snf1 activation is neither caused by elevated autophagy nor mediated by certain transcription factors. (**A**, **B**) Representative images (A) and quantification (B) of FlucSM spGFP in control cells and autophagy-deficient mutants in LG medium. Shown in (A): means ± SEM of spGFP intensity. Unpaired two-tailed *t*-test between control and each mutant. (**C**) Quantification of FlucSM spGFP signals in *Δpdr5* cells treated with DMSO or 80 μM MG132 for 90 min during estradiol induction. Means ± SEM of normalized spGFP intensities are shown. Paired two-tailed *t*-test. (**D**, **E**) Representative images (D) and quantification (E) of FlucSM spGFP in wild-type (*MIG1 MIG2*), *Δmig1*, *Δmig2*, and *Δmig1Δmig2* (*ΔΔ*) cells in HG medium. Shown in (E): means ± SEM of spGFP intensity. Unpaired two-tailed *t*-test between WT and each mutant. (**F**, **G**) Representative images (F) and quantification (G) of FlucSM spGFP in *Δreg1* and double mutant cells in HG medium. Shown in (G): means ± SEM of spGFP intensity. Unpaired two-tailed *t*-test between *Δreg1* and each double mutant. (**H**) Quantification of FlucSM spGFP signals in WT or *Δhap4* cells. Unpaired two-tailed *t*-test between WT and *Δhap4*. Pair two-tailed *t*-test for *Δhap4* cells in different medium. ***P* < 0.01; ****P* < 0.001; ns, not significant, *P* > 0.05. Scale bars, 5 μm.

Figure 3—figure supplement 1

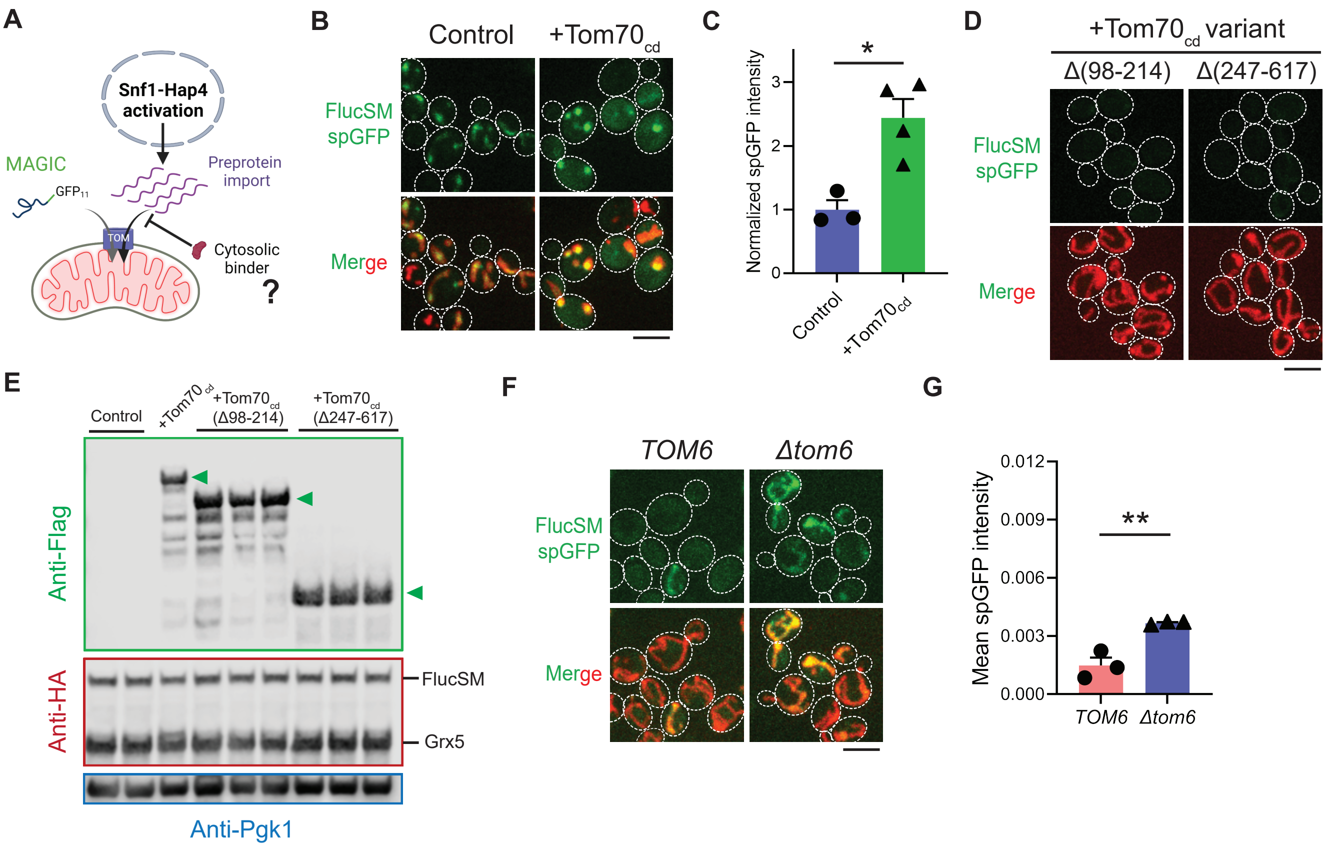

bioRxiv preprint doi: https://doi.org/10.1101/2023.03.29.534670; this version posted August 31, 2023. The copyright holder for this preprint (which was not certified by peer review) is the author/funder, who has granted bioRxiv a license to display the preprint in perpetuity. It is made available under aCC-BY 4.0 International license.

Figure 3—figure supplement 1. Role of Tom70 cytosolic domain and Tom6 in regulating misfolded protein import. (**A**) Working model of preprotein import and misfolded protein import through limited TOM channels. Introducing cytosolic binders of preproteins may release the import capacity for misfolded proteins. (**B**, **C**) Representative images (B) and quantification (C) of FlucSM spGFP in cells with or without $Tom70_{cd}$ overexpression in HG medium. Shown in (C): means ± SEM of normalized spGFP intensity. Unpaired two-tailed *t*-test. (**D**) Representative images of FlucSM spGFP in cells overexpressing truncated Tom70cd variants in LG medium. Quantification is shown in Figure 4C. (**E**) Immunoblots of overexpressed $Tom70_{cd}$-3×Flag variants in lysates of cells that grew in LG medium. Anti-HA panel shows FlucSM-HA-GFP_{11} after 90min estradiol treatment and constitutively expressed Grx5-HA-GFP_{1-10}. Arrowheads indicate intact $Tom70_{cd}$-3×Flag variants. (**F**, **G**) Representative images (F) and quantification (G) of FlucSM spGFP in WT and *Δtom6* cells in LG medium. Shown in (G): means ± SEM of spGFP intensities. Unpaired two-tailed *t*-test. *$P < 0.05$; **$P < 0.01$; ns, not significant, $P > 0.05$. Scale bars, 5 μm.

Figure 4—figure supplement 1

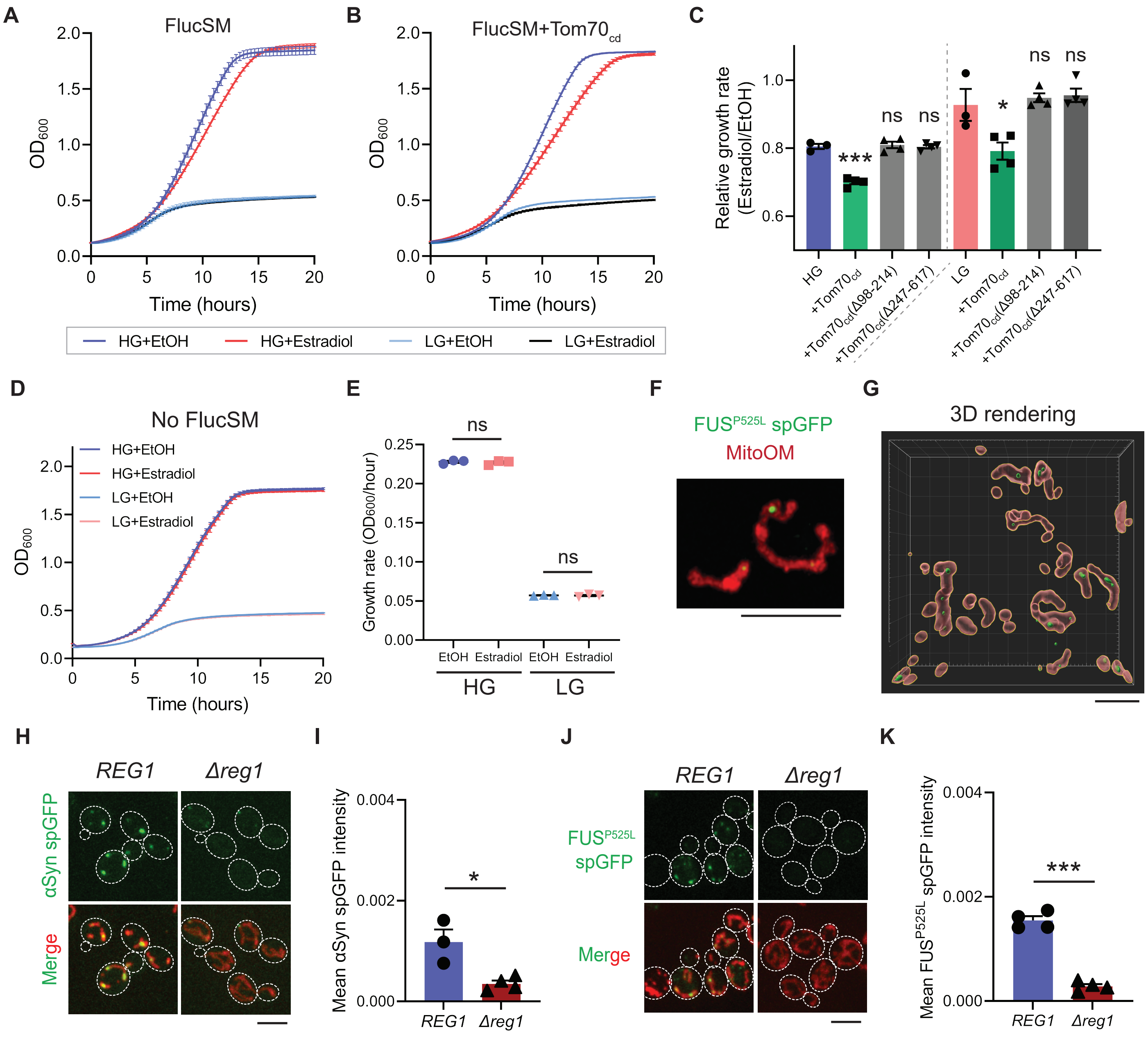

bioRxiv preprint doi: https://doi.org/10.1101/2023.03.29.534670; this version posted August 31, 2023. The copyright holder for this preprint (which was not certified by peer review) is the author/funder, who has granted bioRxiv a license to display the preprint in perpetuity. It is made available under aCC-BY 4.0 International license.

Figure 4—figure supplement 1. Snf1 activation protects against stress associated with FlucSM overexpression and prevents the accumulation of α-synuclein and FUS^{P525L} in yeast mitochondria. (**A**, **B**) Growth curves of wild-type control cells (A) and cells overexpressing Tom70$_{cd}$ (B) with (Estradiol) or without (EtOH) FlucSM expression in HG and LG medium. Fitted growth rates are shown in Figure 4A. (**C**) Relative growth rates of control cells and cells overexpressing Tom70$_{cd}$ variants. Means ± SEM are shown. Unpaired two-tailed *t*-test between control and Tom70$_{cd}$ variants that grew in the same medium (HG or LG). (**D**, **E**) Growth curves (D) and growth rates (E) of BY4741 cells lack of FlucSM spGFP reporter in HG and LG medium. Means ± SEM are shown. Paired two-tailed *t*-test. (**F**, **G**) Representative super-resolution imaging for FUS^{P525L} spGFP signal in mitochondria after 100 min of estradiol induction. Shown in (F): maximum projection image. Shown in (G): 3D rendered image. Mitochondrial OM: Mitochondrial outer membrane labeled with Tom70-mCherry. (**H**-**K**) Representative images (H, J) and quantification (I, K) of α-synuclein (αSyn) spGFP signal FUS^{P525L} spGFP signal in wild-type (*REG1*) and *Δreg1* cells. Shown in (I, K): means ± SEM of spGFP intensity. Unpaired two-tailed *t*-test. HG: 2% glucose; LG: 0.1% glucose plus 3% glycerol. EtOH: ethanol. *$P < 0.05$; **$P < 0.01$; ns, not significant, $P > 0.05$. Scale bars, 5 µm.

bioRxiv preprint doi: https://doi.org/10.1101/2023.03.29.534670; this version posted August 31, 2023. The copyright holder for this preprint (which was not certified by peer review) is the author/funder, who has granted bioRxiv a license to display the preprint in perpetuity. It is made available under aCC-BY 4.0 International license.

Table S1: List of validated MAGIC regulators

(Highlighted in yellow: ribosome-associated genes based on KEGG)

Systematic Name	Standard Name	MAGIC Phenotype
YDR477W	SNF1	Class 1
YML016C	PPZ1	Class 1
YJR120W		Class 1
YOL055C	THI20	Class 1
YKL057C	NUP120	Class 1
YML024W	RPS17A	Class 2
YDR083W	RRP8	Class 2
YCR002C	CDC10	Class 2
YKL143W	LTV1	Class 2
YLL026W	HSP104	Class 2
YPR159W	KRE6	Class 2
YOR096W	RPS7A	Class 2
YMR116C	ASC1	Class 2
YPR057W	BRR1	Class 2
YJR074W	MOG1	Class 2
YCR068W	ATG15	Class 2
YML062C	MFT1	Class 2
YML026C	RPS18B	Class 2
YML013W	UBX2	Class 2
YMR032W	HOF1	Class 2
YNR029C	ZNG1	Class 2
YDL020C	RPN4	Class 2
YER151C	UBP3	Class 2
YMR255W	GFD1	Class 2
YMR307W	GAS1	Class 2
YOR035C	SHE4	Class 2
YOL072W	THP1	Class 2
YDL083C	RPS16B	Class 2
YOR258W	YOR258W	Class 2
YOL129W	VPS68	Class 2
YHR163W	SOL3	Class 2
YLR372W	ELO3	Class 2
YKL191W	DPH2	Class 2
YIR032C	DAL3	Class 2
YBR020W	GAL1	Class 2
YJR145C	RPS4A	Class 2
YDR085C	AFR1	Class 2
YGR019W	UGA1	Class 2
YEL068C		Class 2

bioRxiv preprint doi: https://doi.org/10.1101/2023.03.29.534670; this version posted August 31, 2023. The copyright holder for this preprint (which was not certified by peer review) is the author/funder, who has granted bioRxiv a license to display the preprint in perpetuity. It is made available under aCC-BY 4.0 International license.

YIL112W	HOS4	Class 2
YKL198C	PTK1	Class 2
YER087C-A		Class 2
YJL200C	ACO2	Class 2
YJL160C	PIR5	Class 2
YMR034C	RCH1	Class 2
YGR132C	PHB1	Class 2
YLL033W	IRC19	Class 2
YGR072W	UPF3	Class 2
YGR016W		Class 2
YCR071C	IMG2	Class 2
YER060W	FCY21	Class 2
YER075C	PTP3	Class 2
YGR129W	SYF2	Class 2
YPR146C		Class 2
YEL012W	UBC8	Class 2
YJR113C	RSM7	Class 2
YPL173W	MRPL40	Class 2
YDL057W		Class 2
YBR068C	BAP2	Class 2
YHR200W	RPN10	Class 2
YOR298C-A	MBF1	Class 2
YER056C	FCY2	Class 2
YNL081C	SWS2	Class 2
YGL114W	YGL114W	Class 2
YAR030C		Class 2
YLR053C	NRS1	Class 2
YMR089C	YTA12	Class 2
YBR058C	UBP14	Class 2
YBR175W	SWD3	Class 2
YBR231C	SWC5	Class 2
YDR073W	SNF11	Class 2
YDR115W	MRX14	Class 2
YGR136W	LSB1	Class 2
YGR159C	NSR1	Class 2
YHL033C	RPL8A	Class 2
YHR011W	DIA4	Class 2
YHR143W	DSE2	Class 2
YCL005W	LDB16	Class 2
YCL037C	SRO9	Class 2
YLR131C	ACE2	Class 2
YMR074C	SDD2	Class 2
YKL009W	MRT4	Class 2
YKL128C	PMU1	Class 2

bioRxiv preprint doi: https://doi.org/10.1101/2023.03.29.534670; this version posted August 31, 2023. The copyright holder for this preprint (which was not certified by peer review) is the author/funder, who has granted bioRxiv a license to display the preprint in perpetuity. It is made available under aCC-BY 4.0 International license.

YKL132C	RMA1	Class 2
YGR056W	RSC1	Class 2
YOR125C	CAT5	Class 2
YAL043C-a		Class 2
YLL015W	BPT1	Class 2
YOR235W	IRC13	Class 2
YJL179W	PFD1	Class 2
YLR387C	REH1	Class 2
YLR388W	RPS29A	Class 2
YDR173C	ARG82	Class 2
YGL197W	MDS3	Class 2
YGL194C	HOS2	Class 2
YGL210W	YPT32	Class 2
YPL049C	DIG1	Class 2
YGL085W	LCL3	Class 2
YNL156C	NSG2	Class 2
YKL213C	DOA1	Class 2
YKR042W	UTH1	Class 2
YKR057W	RPS21A	Class 2
YLR065C	SND2	Class 2
YIL043C	CBR1	Class 2
YIL049W	DFG10	Class 2
YIL088C	AVT7	Class 2
YIL054W		Class 2
YOL111C	MDY2	Class 2
YOL122C	SMF1	Class 2
YER091C	MET6	Class 2
YNL316C	PHA2	Class 2
YDL213C	NOP6	Class 2
YDR006C	SOK1	Class 2
YDR025W	RPS11A	Class 2
YBR297W	MAL33	Class 2
YCR025C		Class 2
YML088W	UFO1	Class 2
YNL008C	ASI3	Class 2
YNL010W	PYP1	Class 2
YNR047W	FPK1	Class 2
YBR027C		Class 2
YBR043C	QDR3	Class 2
YML036W	CGI121	Class 2
YPL004C	LSP1	Class 2
YML066C	SMA2	Class 2
YBR133C	HSL7	Class 2
YDL002C	NHP10	Class 2

bioRxiv preprint doi: https://doi.org/10.1101/2023.03.29.534670; this version posted August 31, 2023. The copyright holder for this preprint (which was not certified by peer review) is the author/funder, who has granted bioRxiv a license to display the preprint in perpetuity. It is made available under aCC-BY 4.0 International license.

YBR172C	SMY2	Class 2
YDL021W	GPM2	Class 2
YDR462W	MRPL28	Class 2
YDR500C	RPL37B	Class 2
YGL136C	MRM2	Class 2
YER174C	GRX4	Class 2
YER167W	BCK2	Class 2
YMR221C	FMP42	Class 2
YIL094C	LYS12	Class 2
YGR254W	ENO1	Class 2
YMR257C	PET111	Class 2
YMR278W	PRM15	Class 2
YMR291W	TDA1	Class 2
YMR303C	ADH2	Class 2
YNL303W		Class 2
YNL302C	RPS19B	Class 2
YNL265C	IST1	Class 2
YNL264C	PDR17	Class 2

bioRxiv preprint doi: https://doi.org/10.1101/2023.03.29.534670; this version posted August 31, 2023. The copyright holder for this preprint (which was not certified by peer review) is the author/funder, who has granted bioRxiv a license to display the preprint in perpetuity. It is made available under aCC-BY 4.0 International license.

Table S2: List of yeast strains and plasmids

Strain ID	Genotype	Source	
BY4741	*MATa his3Δ1; leu2Δ0; met15Δ0; ura3Δ0*		
RLY8616	*GRX5-GFP$_{11}$-His3MX6; trp1::P$_{GPD}$-MTS-mCherry-GFP$_{1\text{-}10}$-natMX6*	Ruan, L. et al., 2017.	
RLY8618	*LSG1-GFP$_{11}$-His3MX6; trp1::P$_{GPD}$-MTS-mCherry-GFP$_{1\text{-}10}$-natMX6*	Ruan, L. et al., 2017.	
RLY9798	*LSG1-GFP$_{11}$-His3MX6; trp1::P$_{GPD}$-MTS-mCherry-GFP$_{1\text{-}10}$-natMX6; Δsnf1::kanMX6*	This study	
RLY9799	*LSG1-GFP$_{11}$-His3MX6; trp1::P$_{GPD}$-MTS-mCherry-GFP$_{1\text{-}10}$-natMX6; Δltv1::kanMX6*	This study	
RLY9800	*ura3Δ0::GEM-hphMX6; trp1::P$_{GPD}$-GRX5-HA-GFP$_{1\text{-}10}$-natMX6; HO::P$_{GAL1}$-FlucSM-HA-GFP$_{11}$-His3MX6; TOM70-mCherry-Ura3MX6*	This study	
RLY9801	*ura3Δ0::GEM-hphMX6; trp1::P$_{GPD}$-GRX5-HA-GFP$_{1\text{-}10}$-natMX6; HO::P$_{GAL1}$-FlucSM-HA-GFP$_{11}$-His3MX6; TOM70-mCherry-Ura3MX6; Δreg1::Leu2*	This study	
RLY9802	*trp1::P$_{GPD}$-GRX5-HA-GFP$_{1\text{-}10}$-natMX6; amp::GEM-P$_{GAL1}$-FlucSM-HA-GFP$_{11}$-kanMX6; TOM70-RFP-hphMX6*	This study	
RLY9803	*trp1::P$_{GPD}$-GRX5-HA-GFP$_{1\text{-}10}$-natMX6; amp::GEM-P$_{GAL1}$-FlucSM-HA-GFP$_{11}$-kanMX6; TOM70-mCherry-Ura3MX6*	This study	
RLY9804	*trp1::P$_{GPD}$-GRX5-HA-GFP$_{1\text{-}10}$-natMX6; amp::GEM-P$_{GAL1}$-FlucWT-HA-GFP$_{11}$-kanMX6; TOM70-mCherry-Ura3MX6*	This study	
RLY9805	*trp1::P$_{GPD}$-GRX5-HA-GFP$_{1\text{-}10}$-natMX6; amp::GEM-P$_{GAL1}$-FlucDM-HA-GFP$_{11}$-kanMX6; TOM70-mCherry-Ura3MX6*	This study	
RLY9806	*MIG1-GFP-His3MX6; PUS1-RFP-hphMX6*	This study	
RLY9807	*MIG1-GFP-His3MX6; PUS1-RFP-hphMX6; Δreg1::Leu2*	This study	
RLY9808	*ura3Δ0::P$_{CUP1}$-PIM1-Ura3; GRX5-GFP$_{1\text{-}10}$-natMX6; trp1::P$_{GPD}$-mCherry-Fis1TM-hphMX6; amp::GEM-P$_{GAL1}$-FlucSM-HA-GFP$_{11}$-kanMX6*	This study	
RLY9809	*ura3Δ0::P$_{CUP1}$-pim1^{S974D}-Ura3; GRX5-GFP$_{1\text{-}10}$-natMX6; trp1::P$_{GPD}$-mCherry-Fis1TM-hphMX6; amp::GEM-P$_{GAL1}$-FlucSM-HA-GFP$_{11}$-kanMX6*	This study	
RLY9810	*trp1::P$_{GPD}$-MTS-mCherry-natMX6; amp::GEM-P$_{GAL1}$-FlucSM-HA-GFP$_{11}$-kanMX6*	This study	
RLY9811	*trp1::P$_{GPD}$-MTS-mCherry-natMX6; amp::GEM-P$_{GAL1}$-FlucSM-HA-GFP$_{11}$-kanMX6; Δreg1::His3MX6*	This study	
RLY9812	*ura3Δ0::GEM-hphMX6; trp1::P$_{GPD}$-GRX5-HA-GFP$_{1\text{-}10}$-natMX6; HO::P$_{GAL1}$-FlucSM-HA-GFP$_{11}$-His3MX6; TOM70-mCherry-Ura3MX6; Δreg1::Leu2; Δhap4::kanMX6*	This study	
RLY9813	*trp1::P$_{GPD}$-GRX5-HA-GFP$_{1\text{-}10}$-natMX6; amp::GEM-P$_{GAL1}$-FlucSM-HA-GFP$_{11}$-kanMX6;*	This study	

bioRxiv preprint doi: https://doi.org/10.1101/2023.03.29.534670; this version posted August 31, 2023. The copyright holder for this preprint (which was not certified by peer review) is the author/funder, who has granted bioRxiv a license to display the preprint in perpetuity. It is made available under aCC-BY 4.0 International license.

	TOM70-mCherry-Ura3MX6; HO::P$_{GPD}$-HAP4-hphMX6		
RLY9814	*ura3Δ0::GEM-hphMX6; trp1::P$_{GPD}$-GRX5-HA-GFP$_{1-10}$-natMX6; HO::P$_{GAL1}$-FlucSM-HA-GFP$_{11}$-His3MX6; TOM70-mCherry-Ura3MX6; Δatg1::kanMX6*	This study	
RLY9815	*ura3Δ0::GEM-hphMX6; trp1::P$_{GPD}$-GRX5-HA-GFP$_{1-10}$-natMX6; HO::P$_{GAL1}$-FlucSM-HA-GFP$_{11}$-His3MX6; TOM70-mCherry-Ura3MX6; Δatg15::kanMX6*	This study	
RLY9816	*ura3Δ0::GEM-hphMX6; trp1::P$_{GPD}$-GRX5-HA-GFP$_{1-10}$-natMX6; HO::P$_{GAL1}$-FlucSM-HA-GFP$_{11}$-His3MX6; TOM70-mCherry-Ura3MX6; Δmig1::kanMX6*	This study	
RLY9817	*ura3Δ0::GEM-hphMX6; trp1::P$_{GPD}$-GRX5-HA-GFP$_{1-10}$-natMX6; HO::P$_{GAL1}$-FlucSM-HA-GFP$_{11}$-His3MX6; TOM70-mCherry-Ura3MX6; Δmig2::Leu2*	This study	
RLY9818	*ura3Δ0::GEM-hphMX6; trp1::P$_{GPD}$-GRX5-HA-GFP$_{1-10}$-natMX6; HO::P$_{GAL1}$-FlucSM-HA-GFP$_{11}$-His3MX6; TOM70-mCherry-Ura3MX6; Δmig1::kanMX6; Δmig2::Leu2*	This study	
RLY9819	*ura3Δ0::GEM-hphMX6; trp1::P$_{GPD}$-GRX5-HA-GFP1-10-natMX6; HO::PGAL1-FlucSM-HA-GFP11-His3MX6; TOM70-mCherry-Ura3MX6; Δreg1::Leu2; Δcat8::kanMX6*	This study	
RLY9820	*ura3Δ0::GEM-hphMX6; trp1::P$_{GPD}$-GRX5-HA-GFP1-10-natMX6; HO::PGAL1-FlucSM-HA-GFP11-His3MX6; TOM70-mCherry-Ura3MX6; Δreg1::Leu2; Δsip4::kanMX6*	This study	
RLY9821	*ura3Δ0::GEM-hphMX6; trp1::P$_{GPD}$-GRX5-HA-GFP1-10-natMX6; HO::PGAL1-FlucSM-HA-GFP11-His3MX6; TOM70-mCherry-Ura3MX6; Δreg1::Leu2; Δrds2::kanMX6*	This study	
RLY9822	*ura3Δ0::GEM-hphMX6; trp1::P$_{GPD}$-GRX5-HA-GFP1-10-natMX6; HO::PGAL1-FlucSM-HA-GFP11-His3MX6; TOM70-mCherry-Ura3MX6; Δreg1::Leu2; Δadr1::kanMX6*	This study	
RLY9823	*trp1::P$_{GPD}$-GRX5-HA-GFP$_{1-10}$-natMX6; amp::GEM-P$_{GAL1}$-FlucSM-HA-GFP$_{11}$-kanMX6; TOM70-mCherry-Ura3MX6; HO::P$_{GPD}$-tom20$_{cd}$-hphMX6*	This study	
RLY9824	*trp1::P$_{GPD}$-GRX5-HA-GFP$_{1-10}$-natMX6; amp::GEM-P$_{GAL1}$-FlucSM-HA-GFP$_{11}$-kanMX6; TOM70-mCherry-Ura3MX6; HO::P$_{GPD}$-tom22$_{cd}$-hphMX6*	This study	
RLY9825	*ura3Δ0::GEM-hphMX6; trp1::P$_{GPD}$-GRX5-HA-GFP$_{1-10}$-natMX6; HO::P$_{GAL1}$-FlucSM-HA-GFP$_{11}$-His3MX6; TOM70-mCherry-Ura3MX6; amp::P$_{GPD}$-tom70$_{cd}$-3xFLAG-kanMX6*	This study	

bioRxiv preprint doi: https://doi.org/10.1101/2023.03.29.534670; this version posted August 31, 2023. The copyright holder for this preprint (which was not certified by peer review) is the author/funder, who has granted bioRxiv a license to display the preprint in perpetuity. It is made available under aCC-BY 4.0 International license.

RLY9826	*ura3Δ0::GEM-hphMX6; trp1::P$_{GPD}$-GRX5-HA-GFP$_{1-10}$-natMX6; HO::P$_{GAL1}$-FlucSM-HA-GFP$_{11}$-His3MX6; TOM70-mCherry-Ura3MX6; amp::P$_{GPD}$-tom70$_{cd}$(Δ98-214)-3xFLAG-kanMX6*	This study	
RLY9827	*ura3Δ0::GEM-hphMX6; trp1::P$_{GPD}$-GRX5-HA-GFP$_{1-10}$-natMX6; HO::P$_{GAL1}$-FlucSM-HA-GFP$_{11}$-His3MX6; TOM70-mCherry-Ura3MX6; amp::P$_{GPD}$-tom70$_{cd}$(Δ247-617)-3xFLAG-kanMX6*	This study	
RLY9828	*ura3Δ0::GEM-hphMX6; trp1::P$_{GPD}$-GRX5-HA-GFP$_{1-10}$-natMX6; HO::P$_{GAL1}$-FlucSM-HA-GFP$_{11}$-His3MX6; trp1::P$_{GPD}$-mCherry-Fis1TM-kanMX6*	This study	
RLY9829	*ura3Δ0::GEM-hphMX6; trp1::P$_{GPD}$-GRX5-HA-GFP$_{1-10}$-natMX6; HO::P$_{GAL1}$-FlucSM-HA-GFP$_{11}$-His3MX6; trp1::P$_{GPD}$-mCherry-Fis1TM-kanMX6; Δtom70::Ura3MX6*	This study	
RLY9830	*ura3Δ0::GEM-hphMX6; trp1::P$_{GPD}$-GRX5-HA-GFP$_{1-10}$-natMX6; HO::P$_{GAL1}$-FlucSM-HA-GFP$_{11}$-His3MX6; trp1::P$_{GPD}$-mCherry-Fis1TM-kanMX6; Δtom71::Leu2*	This study	
RLY9831	*ura3Δ0::GEM-hphMX6; trp1::P$_{GPD}$-GRX5-HA-GFP$_{1-10}$-natMX6; HO::P$_{GAL1}$-FlucSM-HA-GFP$_{11}$-His3MX6; trp1::P$_{GPD}$-mCherry-Fis1TM-kanMX6; Δtom70::Ura3MX6; Δtom71::Leu2*	This study	
RLY9832	*ura3Δ0::GEM-hphMX6; trp1::P$_{GPD}$-GRX5-HA-GFP$_{1-10}$-natMX6; HO::P$_{GAL1}$-FlucSM-HA-GFP$_{11}$-His3MX6; TOM70-mCherry-Ura3MX6; amp::P$_{GPD}$-tom70$_{cd}$-3xFLAG-kanMX6; Δtom6::Leu2*	This study	
RLY9833	*ura3Δ0::GEM-hphMX6; trp1::P$_{GPD}$-GRX5-HA-GFP$_{1-10}$-natMX6; HO::P$_{GAL1}$-FlucSM-HA-GFP$_{11}$-His3MX6*	This study	
RLY9834	*ura3Δ0::GEM-hphMX6; trp1::P$_{GPD}$-GRX5-HA-GFP$_{1-10}$-natMX6; HO::P$_{GAL1}$-FlucSM-HA-GFP$_{11}$-His3MX6; amp::P$_{GPD}$-tom70$_{cd}$-3xFLAG-kanMX6*	This study	
RLY9835	*ura3Δ0::P$_{GPD}$-a-Synuclein-HA-GFP$_{11}$-His3MX6; GRX5-GFP$_{1-10}$-natMX6; TOM70-mCherry-Ura3MX6*	This study	
RLY9836	*ura3Δ0::P$_{GPD}$-a-Synuclein-HA-GFP$_{11}$-His3MX6; GRX5-GFP$_{1-10}$-natMX6; TOM70-mCherry-Ura3MX6; Δreg1::Leu2*	This study	
RLY9837	*ura3Δ0::P$_{GPD}$-a-Synuclein-HA-GFP11-His3MX6; GRX5-GFP1-10-natMX6; TOM70-mCherry-Ura3MX6; trp1::P$_{GPD}$-tom70$_{cd}$-3xFLAG-kanMX6*	This study	
RLY9838	*ura3Δ0::P$_{GPD}$-a-Synuclein-HA-GFP$_{11}$-His3MX6; GRX5-GFP$_{1-10}$-natMX6*	This study	
RLY9839	*ura3Δ0::P$_{GPD}$-a-Synuclein-HA-GFP11-His3MX6; GRX5-GFP1-10-natMX6; trp1::P$_{GPD}$-tom70$_{cd}$-3xFLAG-kanMX6*	This study	

bioRxiv preprint doi: https://doi.org/10.1101/2023.03.29.534670; this version posted August 31, 2023. The copyright holder for this preprint (which was not certified by peer review) is the author/funder, who has granted bioRxiv a license to display the preprint in perpetuity. It is made available under aCC-BY 4.0 International license.

RLY9840	*trp1::P$_{GPD}$-GRX5-HA-GFP$_{1-10}$-natMX6; TOM70-mCherry-Ura3MX6; amp::GEM-P$_{GAL1}$-FUS^{P525L}-HA-GFP$_{11}$-kanMX6*	This study	
RLY9841	*ura3Δ0::GEM-hphMX6; trp1::P$_{GPD}$-GRX5-HA-GFP$_{1-10}$-natMX6; HO::P$_{GAL1}$-FUS^{P525L}-HA-GFP$_{11}$-His3MX6; TOM70-mCherry-Ura3MX6*	This study	
RLY9842	*ura3Δ0::GEM-hphMX6; trp1::P$_{GPD}$-GRX5-HA-GFP$_{1-10}$-natMX6; HO::P$_{GAL1}$-FUS^{P525L}-HA-GFP$_{11}$-His3MX6; TOM70-mCherry-Ura3MX6; Δreg1::Leu2*	This study	
RLY9843	*ura3Δ0::GEM-hphMX6; trp1::P$_{GPD}$-GRX5-HA-GFP$_{1-10}$-natMX6; HO::P$_{GAL1}$-FUS^{P525L}-HA-GFP$_{11}$-His3MX6; TOM70-mCherry-Ura3MX6; amp::P$_{GPD}$-tom70$_{cd}$-3xFLAG-kanMX6*	This study	
RLY9844	*trp1::P$_{GPD}$-GRX5-HA-GFP$_{1-10}$-natMX6; amp::GEM-P$_{GAL1}$-FUS^{P525L}-HA-GFP$_{11}$-kanMX6*	This study	
RLY9845	*trp1::P$_{GPD}$-GRX5-HA-GFP$_{1-10}$-natMX6; amp::GEM-P$_{GAL1}$-FUS^{P525L}-HA-GFP$_{11}$-kanMX6; trp1::P$_{GPD}$-tom70$_{cd}$-3xFLAG-kanMX6*	This study	
RLY9846	*ura3Δ0::GEM-hphMX6; trp1::P$_{GPD}$-GRX5-HA-GFP$_{1-10}$-natMX6; HO::P$_{GAL1}$-FlucSM-HA-GFP$_{11}$-His3MX6; TOM70-mCherry-Ura3MX6; Δpdr5::kanMX6*	This study	
RLY9847	*ura3Δ0::GEM-hphMX6; trp1::P$_{GPD}$-GRX5-HA-GFP$_{1-10}$-natMX6; HO::P$_{GAL1}$-FlucSM-HA-GFP$_{11}$-His3MX6; TOM70-mCherry-Ura3MX6; Δhap4::kanMX6*	This study	

**Plasmid ID**	**Construct**	**Vector type**	**Source**
RLB918	*TRP1::P$_{GPD}$-MTS-mCherry-GFP$_{1-10}$-natMX6*	Yeast expression	Ruan, L. et al., 2017.
RLB919	*TRP1::P$_{GPD}$-Grx5-HA-GFP$_{1-10}$-natMX6*	Yeast expression	Ruan, L. et al., 2017.
pJW1663	*GEM-P$_{GAL1}$-GFP-kanMX6*	Yeast expression	Costa, E.A. et al. 2018.
RLB1050	*TRP1::P$_{GPD}$-mCherry-Fis1TM-KanMX6*	Yeast expression	Ruan, L. et al., 2017.
RLB1051	*GEM-P$_{GAL1}$-FlucSM-HA-GFP$_{11}$-KanMX6*	Yeast expression	This study
RLB1052	*GEM-P$_{GAL1}$-FlucWT-HA-GFP$_{11}$-KanMX6*	Yeast expression	This study
RLB1053	*GEM-P$_{GAL1}$-FlucDM-HA-GFP$_{11}$-KanMX6*	Yeast expression	This study
RLB1054	*GEM-P$_{GAL1}$-FUS^{P525L}-HA-GFP$_{11}$-KanMX6*	Yeast expression	This study
RLB1055	*pRS316-P$_{CUP1}$-PIM1-Ura3*	Yeast expression	Nitika et al. 2022
RLB1056	*pRS316-P$_{CUP1}$-pim1^{S974D}-Ura3*	Yeast expression	Nitika et al. 2022

bioRxiv preprint doi: https://doi.org/10.1101/2023.03.29.534670; this version posted August 31, 2023. The copyright holder for this preprint (which was not certified by peer review) is the author/funder, who has granted bioRxiv a license to display the preprint in perpetuity. It is made available under aCC-BY 4.0 International license.

RLB1057	*pRS313-HO(homology)-P$_{GAL1}$-FlucSM-HA-GFP$_{11}$-His3MX6-HO(homology)*	Yeast expression	This study
RLB1058	*pRS313-HO(homology)-P$_{GAL1}$-FUS^{P525L}-HA-GFP$_{11}$-His3MX6-HO(homology)*	Yeast expression	This study
RLB1059	*pRS316-HO(homology)-P$_{GPD}$-HAP4-hphMX6-HO(homology)*	Yeast expression	This study
RLB1060	*TRP1::P$_{GPD}$-tom70$_{cd}$-3xFLAG-KanMX6*	Yeast expression	This study
RLB1061	*TRP1::P$_{GPD}$-tom70$_{cd}$(Δ98-214)-3xFLAG-KanMX6*	Yeast expression	This study
RLB1062	*TRP1::P$_{GPD}$-tom70$_{cd}$(Δ247-617)-3xFLAG-KanMX6*	Yeast expression	This study
RLB1063	*pRS316-HO(homology)-P$_{GPD}$-tom20$_{cd}$-hphMX6-HO(homology)*	Yeast expression	This study
RLB1064	*pRS316-HO(homology)-P$_{GPD}$-tom22$_{cd}$-hphMX6-HO(homology)*	Yeast expression	This study
RLB1065	*P$_{GPD}$-a-Synuclein-HA-GFP$_{11}$-His3MX6*	Yeast expression	This study
RLB1066	*P$_{CMV}$-FUS^{P525L}-HA-GFP$_{11}$*	Mammalian expression	Ruan, L. et al., 2017.
RLB912	*P$_{CMV}$-MTS-mCherry-GFP$_{1-10}$*	Mammalian expression	Ruan, L. et al., 2017.
RLB914	*P$_{CMV}$-FlucDM-HA-GFP$_{11}$*	Mammalian expression	Ruan, L. et al., 2017.
RLB916	*P$_{CMV}$-GST-HA-GFP$_{11}$*	Mammalian expression	Ruan, L. et al., 2017.
