# Supplementary material for: Metabolic regulation of misfolded protein import into mitochondria
Source: bioRxiv. 2023 Mar 29:2023.03.29.534670. Preprint. [Version 1] doi: 10.1101/2023.03.29.534670 (PMC10081186; doi:10.1101/2023.03.29.534670)
Supplement: Supplementary file 1 [file NIHPP2023.03.29.534670v1-supplement-1.pdf]

bioRxiv preprint doi: https://doi.org/10.1101/2023.03.29.534670; this version posted March 29, 2023. The copyright holder for this preprint (which was not certified by peer review) is the author/funder, who has granted bioRxiv a license to display the preprint in perpetuity. It is made available under aCC-BY 4.0 International license.

Supplementary Materials

Table S1: List of validated MAGIC regulators

Table S2: List of yeast strains and plasmids

Movie S1: 3D reconstructed SIM images showing FlucSM spGFP inside mitochondria after 90 min estradiol treatment. Mitochondria outer membrane was labeled with Tom70-mCherry.

Movie S2: 3D reconstructed SIM images showing FUS^{P525L} spGFP inside mitochondria after 100 min estradiol treatment. Mitochondria outer membrane was labeled with Tom70-mCherry.

bioRxiv preprint doi: https://doi.org/10.1101/2023.03.29.534670; this version posted March 29, 2023. The copyright holder for this preprint (which was not certified by peer review) is the author/funder, who has granted bioRxiv a license to display the preprint in perpetuity. It is made available under aCC-BY 4.0 International license.

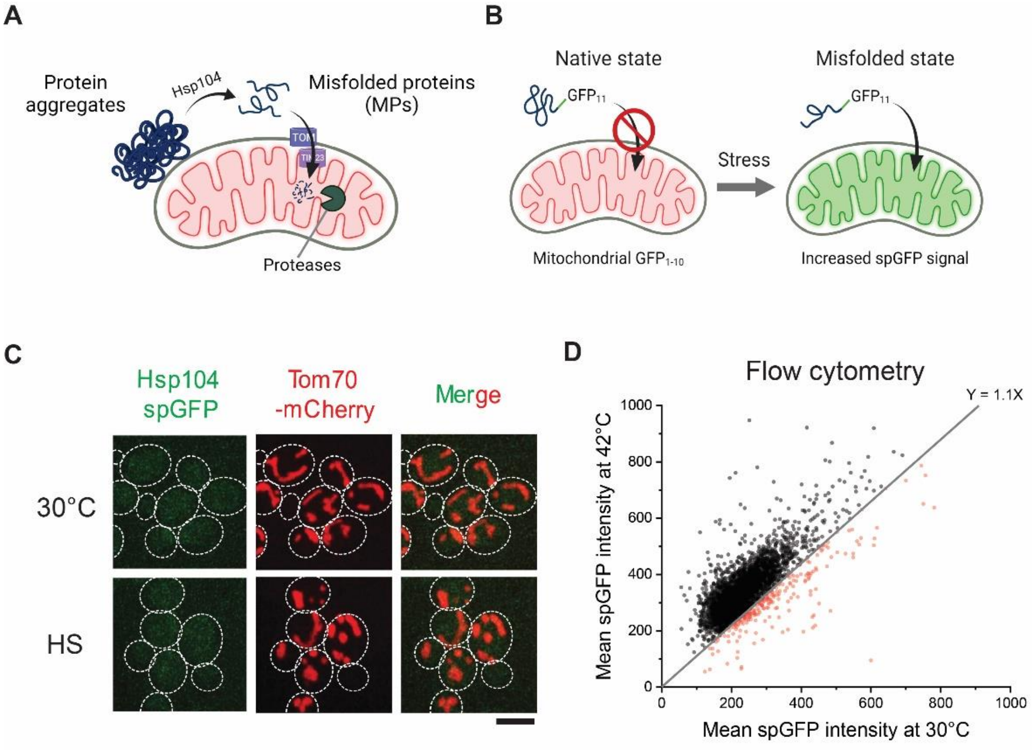

Figure 1—figure supplement 1. Schematics of MAGIC pathway and spGFP-based imaging in the whole-genome screen in yeast. (**A**) Schematic diagram of MAGIC involving the import of cytosolic misfolded proteins through mitochondrial import machineries and the subsequent degradation under proteotoxic stresses (heat shock or overload of MPs). (**B**) Schematic diagram of spGFP reporter. (**C**) Hsp104 spGFP in mitochondria at 30 °C and after heat shock (HS) at 42 °C for 30 min. Endogenous Hsp104 was tagged with GFP$_{11}$, while GFP$_{1-10}$ was constitutively targeted to the mitochondrial matrix by linking to matrix protein Grx5. Similar results were obtained by using MTS-mCherry-GFP$_{1-10}$ (*7*). 3 biological repeats, 63 cells (30 °C) and 220 cells (HS) imaged. Scale bar, 5 μm. (**D**) Flow cytometry readouts of yeast knockout mutants at 30 °C and after 42 °C HS. Red dots indicate mutants that failed to show an increase in Lsg1 spGFP after HS.

bioRxiv preprint doi: https://doi.org/10.1101/2023.03.29.534670; this version posted March 29, 2023. The copyright holder for this preprint (which was not certified by peer review) is the author/funder, who has granted bioRxiv a license to display the preprint in perpetuity. It is made available under aCC-BY 4.0 International license.

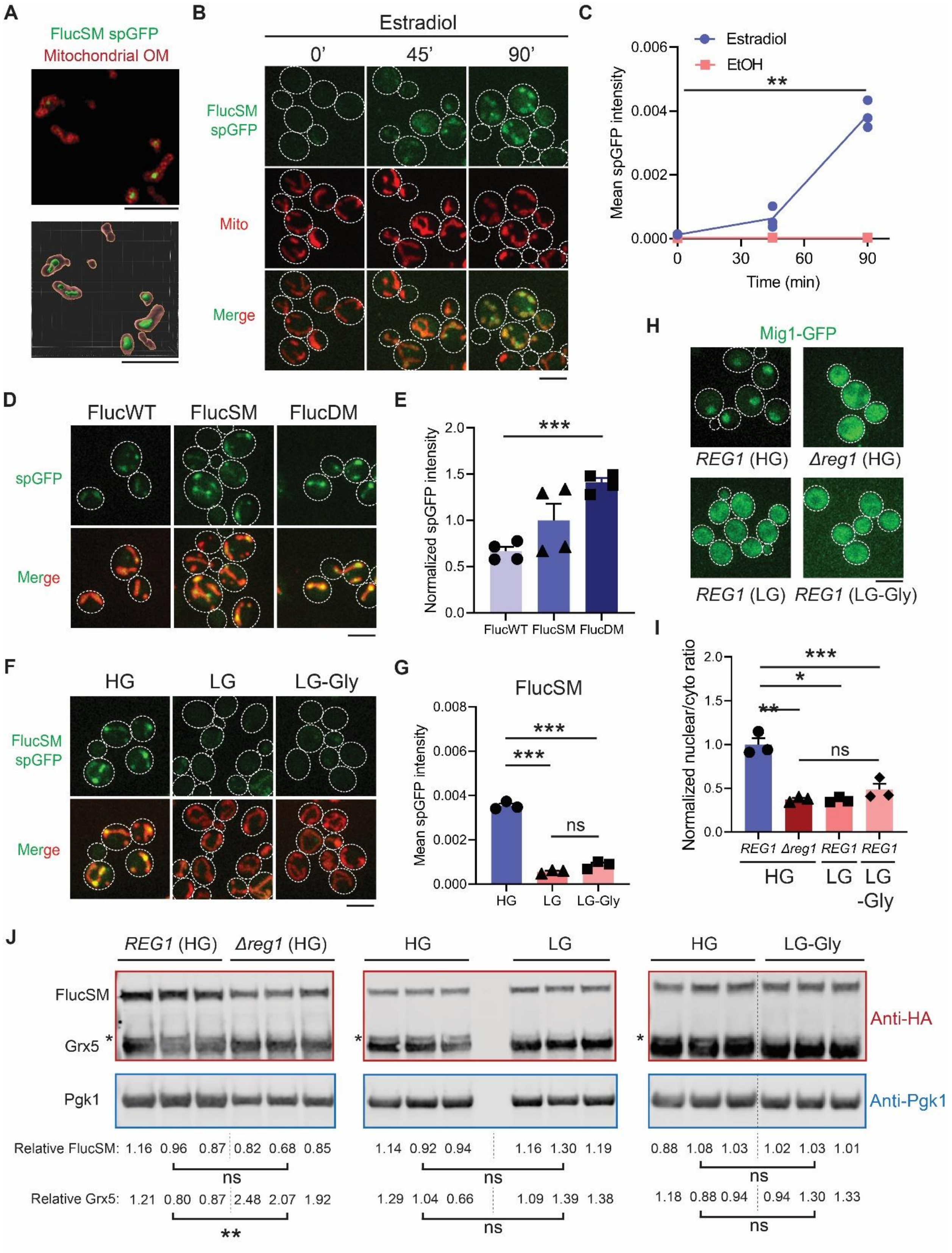

bioRxiv preprint doi: https://doi.org/10.1101/2023.03.29.534670; this version posted March 29, 2023. The copyright holder for this preprint (which was not certified by peer review) is the author/funder, who has granted bioRxiv a license to display the preprint in perpetuity. It is made available under aCC-BY 4.0 International license.

Figure 1—figure supplement 2. Snf1 regulates the accumulation of misfolded proteins in mitochondria after acute overexpression of FlucSM. (**A**) Representative super-resolution imaging for FlucSM spGFP signal in mitochondria after 90 min estradiol induction. Top, maximum projection. Bottom, 3D rendering. OM: outer membrane labeled by Tom70-mCherry. (**B**, **C**) Representative images (B) and quantification (C) of time-dependent accumulation of FlucSM spGFP signal in mitochondria at 30 °C after estradiol or ethanol treatment in HG medium. Shown in (B): Top, FlucSM spGFP; Middle: mitochondria labeled with Tom70-mCherry; Bottom: merged images. Shown in (C): means ± SEM of spGFP intensity. Paired two-tailed *t*-test comparing 0 min and 90 min estradiol treatment. (**D**, **E**) Representative images (D) and quantification (E) of FlucWT, FlucSM, and FlucDM spGFP after 90 min estradiol treatment in HG medium. Shown in (E): means ± SEM of normalized spGFP intensity. Unpaired two-tailed t-test between FlucWT and FlucDM. (**F**, **G**) Representative images (F) and quantification (G) of FlucSM spGFP in cells that grew in HG, LG, and LG-Gly media. Shown in (G): means ± SEM of spGFP intensity. Paired two-tailed *t*-test. (**H**, **I**) Representative images (H) and quantification (I) of the nuclear-cytoplasmic translocation of Mig1-GFP. Shown in (I): means ± SEM of normalized Mig-GFP nuclear-cytoplasmic ratio. Paired (WT in HG vs. LG or LG-Gly) or unpaired (WT vs. *Δreg1* in HG) two-tailed *t*-test, and one-way ANOVA for comparing *Δreg1* in HG, WT in LG, and LG-Gly). (**J**) Immunoblots of FlucSM-HA-GFP_{11} and Grx5-HA-$GFP_{1\text{-}10}$ in cell lysates of Snf1-inactive and Snf1-active cells. Relative FlucSM levels (FlucSM/Pgk1) and relative Grx5 levels (matured Grx5/Pgk1) of three biological repeats are shown. Paired (HG vs. LG or LG-Gly) or unpaired (WT vs. *Δreg1* in HG) two-tailed *t*-test. Asterisk indicates the precursor species of Grx5-HA-$GFP_{1\text{-}10}$ that are excluded from quantification. HG: 2% glucose; LG: 0.1% glucose plus 3% glycerol; LG-Gly: 0.1% glucose. EtOH: ethanol. *$P < 0.05$; **$P < 0.01$; ***$P < 0.001$; ns, not significant, $P > 0.05$. Scale bars, 5 μm.

bioRxiv preprint doi: https://doi.org/10.1101/2023.03.29.534670; this version posted March 29, 2023. The copyright holder for this preprint (which was not certified by peer review) is the author/funder, who has granted bioRxiv a license to display the preprint in perpetuity. It is made available under aCC-BY 4.0 International license.

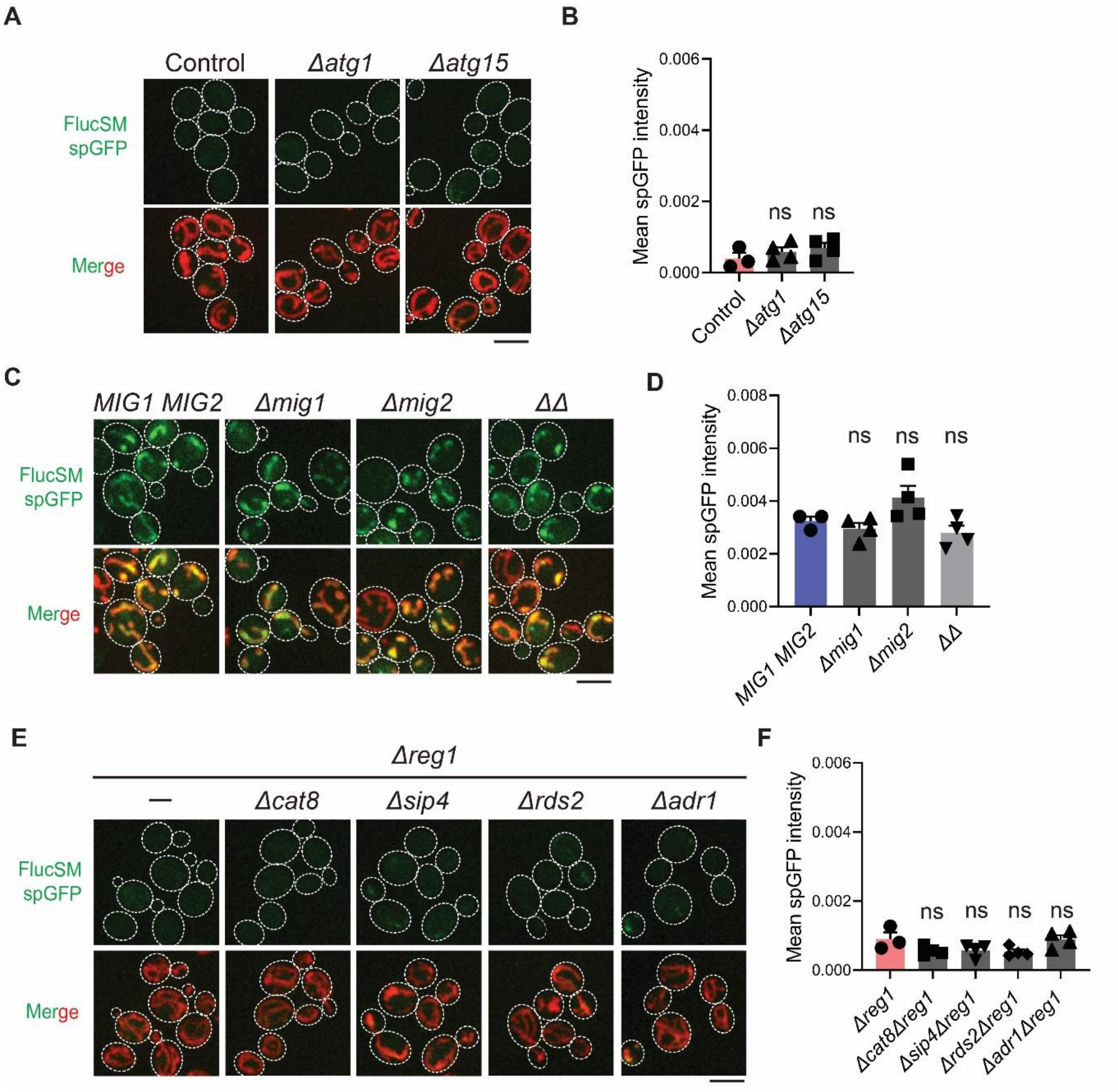

Figure 2—figure supplement 1. Reduced accumulation of misfolded proteins in mitochondria in Snf1-active cells is neither caused by elevated autophagy nor mediated by certain transcription factors. (**A**, **B**) Representative images (A) and quantification (B) of FlucSM spGFP in control cells and autophagy-deficient mutants in LG medium. Shown in (A): means ± SEM of spGFP intensity. Unpaired two-tailed *t*-test between control and each mutant. (**C**, **D**) Representative images (C) and quantification (D) of FlucSM spGFP in wild-type (*MIG1 MIG2*), *Δmig1*, *Δmig2*, and *Δmig1Δmig2* (*ΔΔ*) cells in HG medium. Shown in (D): means ± SEM of spGFP intensity. Unpaired two-tailed *t*-test between WT and each mutant. (**E**, **F**) Representative images (E) and quantification (F) of FlucSM spGFP in *Δreg1* and double mutant cells in HG medium. Shown in (F): means ± SEM of spGFP intensity. Unpaired two-tailed *t*-test between *Δreg1* and each double mutant. ns, not significant, $P > 0.05$. Scale bars, 5 μm.

bioRxiv preprint doi: https://doi.org/10.1101/2023.03.29.534670; this version posted March 29, 2023. The copyright holder for this preprint (which was not certified by peer review) is the author/funder, who has granted bioRxiv a license to display the preprint in perpetuity. It is made available under aCC-BY 4.0 International license.

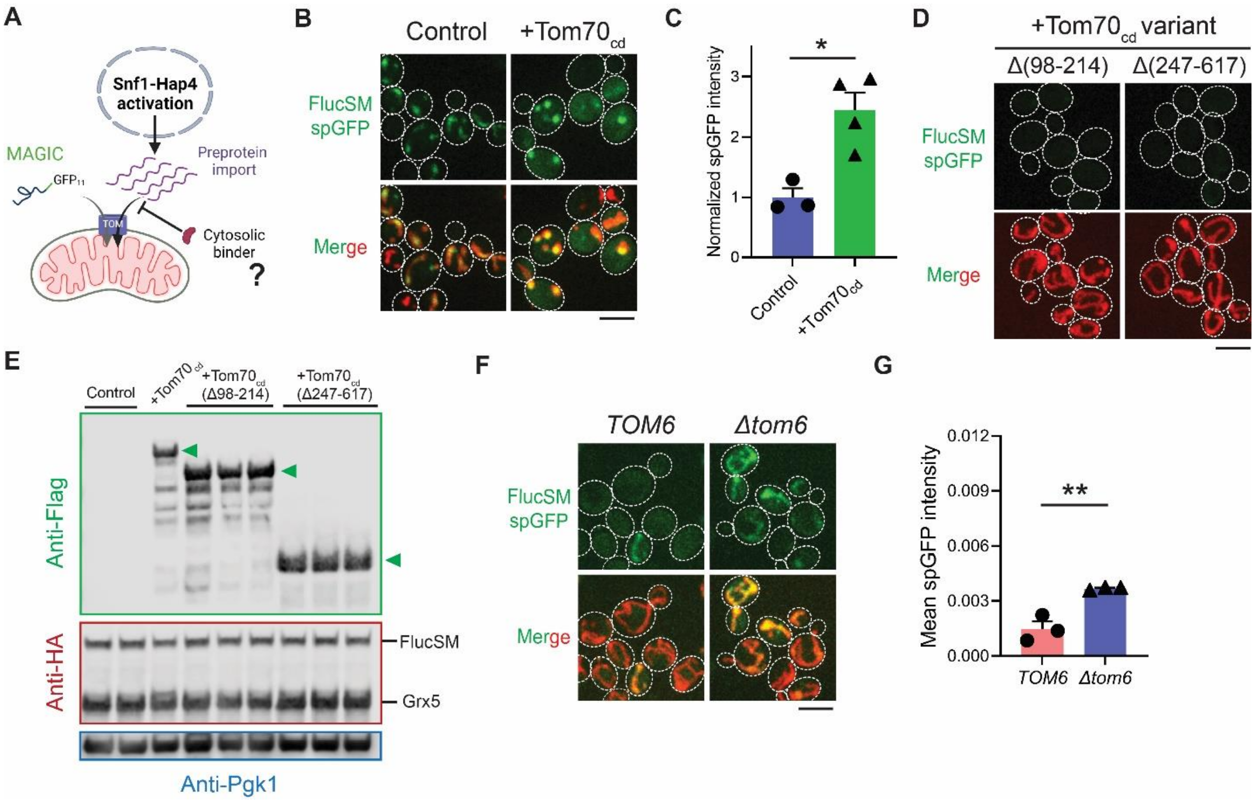

Figure 3—figure supplement 1. Role of Tom70 cytosolic domain and Tom6 in regulating misfolded protein import. (**A**) Working model of preprotein import and misfolded protein import through limited TOM channels. Introducing cytosolic binders of preproteins may release the import capacity for misfolded proteins. (**B**, **C**) Representative images (B) and quantification (C) of FlucSM spGFP in cells with or without $Tom70_{cd}$ overexpression in HG medium. Shown in (C): means ± SEM of normalized spGFP intensity. Unpaired two-tailed *t*-test. (**D**) Representative images of FlucSM spGFP in cells overexpressing truncated Tom70cd variants in LG medium. Quantification is shown in Figure 4C. (**E**) Immunoblots of overexpressed $Tom70_{cd}$-3×Flag variants in lysates of cells that grew in LG medium. Anti-HA panel shows FlucSM-HA-GFP_{11} after 90min estradiol treatment and constitutively expressed Grx5-HA-$GFP_{1\text{-}10}$. Arrowheads indicate intact $Tom70_{cd}$-3×Flag variants. (**F**, **G**) Representative images (F) and quantification (G) of FlucSM spGFP in WT and *Δtom6* cells in LG medium. Shown in (G): means ± SEM of spGFP intensity. Unpaired two-tailed *t*-test. *$P < 0.05$; **$P < 0.01$; ns, not significant, $P > 0.05$. Scale bars, 5 μm.

bioRxiv preprint doi: https://doi.org/10.1101/2023.03.29.534670; this version posted March 29, 2023. The copyright holder for this preprint (which was not certified by peer review) is the author/funder, who has granted bioRxiv a license to display the preprint in perpetuity. It is made available under aCC-BY 4.0 International license.

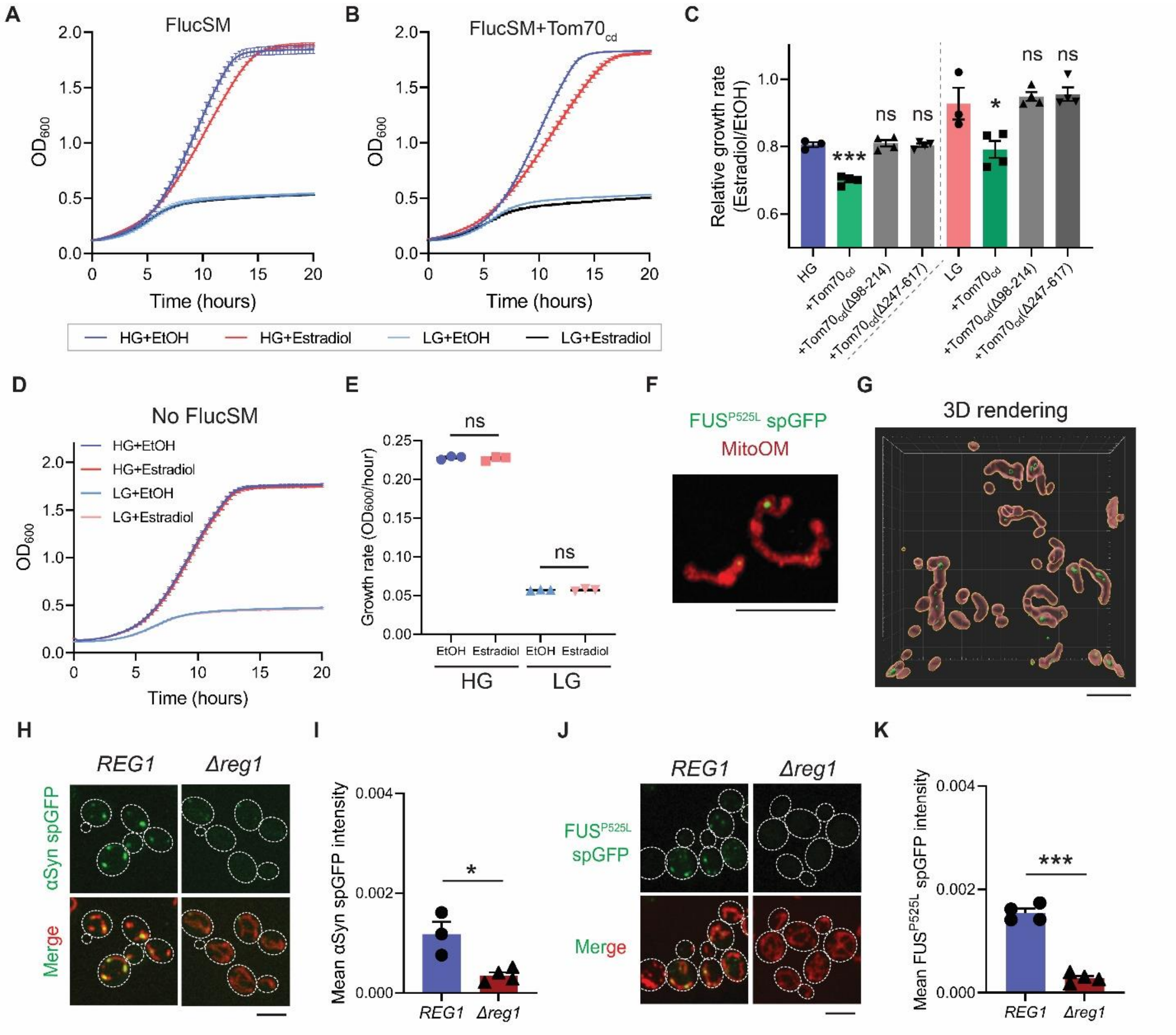

Figure 4—figure supplement 1. Snf1 activation protects against stress associated with FlucSM overexpression and prevents the accumulation of α-synuclein and FUS^{P525L} in yeast mitochondria. (**A**, **B**) Growth curves of wild-type control cells (A) and cells overexpressing Tom70$_{cd}$ (B) with (Estradiol) or without (EtOH) FlucSM expression in HG and LG medium. Fitted growth rates are shown in Figure 4A. (**C**) Relative growth rates of control cells and cells overexpressing Tom70$_{cd}$ variants. Means ± SEM are shown. Unpaired two-tailed *t*-test between control and Tom70$_{cd}$ variants that grew in the same medium (HG or LG). (**D**, **E**) Growth curves (D) and growth rates (E) of BY4741 cells lack of FlucSM spGFP reporter in HG and LG medium. Means ± SEM are shown. Paired two-tailed *t*-test. (**F**, **G**) Representative super-resolution imaging for FUS^{P525L} spGFP signal in mitochondria after 100 min of estradiol induction. Shown in (F): maximum projection image. Shown in (G): 3D rendered image. Mitochondrial OM: Mitochondrial outer membrane labeled with Tom70-mCherry. (**H**-**K**) Representative images (H, J) and quantification (I, K) of α-synuclein (αSyn) spGFP signal FUS^{P525L} spGFP signal in wild-type (*REG1*) and *Δreg1* cells. Shown in (I, K): means ± SEM of spGFP intensity. Unpaired two-tailed *t*-test. HG: 2% glucose; LG: 0.1% glucose plus 3% glycerol. EtOH: ethanol. *P < 0.05; **P < 0.01; ns, not significant, P > 0.05. Scale bars, 5 μm.
